# Leukocytes mediate disease pathogenesis in the *Ndufs4*(KO) mouse model of Leigh syndrome

**DOI:** 10.1172/jci.insight.156522

**Published:** 2022-03-08

**Authors:** Julia C. Stokes, Rebecca L. Bornstein, Katerina James, Kyung Yeon Park, Kira A. Spencer, Katie Vo, John C. Snell, Brittany M. Johnson, Philip G. Morgan, Margaret M. Sedensky, Nathan A. Baertsch, Simon C. Johnson

**Affiliations:** 1 Center for Integrative Brain Research, Seattle Children’s Research Institute, Seattle, Washington, USA.; 2 Department of Laboratory Medicine and Pathology,; 3 Department of Neurology,; 4 Department of Anesthesiology and Pain Medicine, and; 5 Department of Pediatrics, University of Washington, Seattle, Washington, USA.

**Keywords:** Inflammation, Neuroscience, Genetic diseases, Mitochondria, Mouse models

## Abstract

Symmetric, progressive, necrotizing lesions in the brainstem are a defining feature of Leigh syndrome (LS). A mechanistic understanding of the pathogenesis of these lesions has been elusive. Here, we report that leukocyte proliferation is causally involved in the pathogenesis of LS. Depleting leukocytes with a colony-stimulating factor 1 receptor inhibitor disrupted disease progression, including suppression of CNS lesion formation and a substantial extension of survival. Leukocyte depletion rescued diverse symptoms, including seizures, respiratory center function, hyperlactemia, and neurologic sequelae. These data reveal a mechanistic explanation for the beneficial effects of mTOR inhibition. More importantly, these findings dramatically alter our understanding of the pathogenesis of LS, demonstrating that immune involvement is causal in disease. This work has important implications for the mechanisms of mitochondrial disease and may lead to novel therapeutic strategies.

## Introduction

Grouped, genetic mitochondrial diseases (GMDs) are the most common cause of heritable metabolic disease and among the most common causes of pediatric neurologic dysfunction (1, 2). GMDs are genetically and clinically heterogeneous, grouping into clinically defined syndromes (2). Subacute necrotizing encephalopathy, or Leigh syndrome (LS), is the most common form of pediatric GMD. LS presents as a multisystem disorder with metabolic, neurologic, and musculoskeletal symptoms (3). Patients with LS are often born healthy, with symptoms presenting in the first years of life. Symmetric, progressive, necrotizing brainstem lesions are a defining feature of LS. A mechanistic understanding of these lesions has been elusive, and no effective clinical interventions exist. Inhibition of the mechanistic target of rapamycin (mTOR) attenuates disease in the *Ndufs4*(KO) mouse model of LS (4, 5), and mTOR inhibitors appears to benefit some patients (6, 7). The precise mechanism(s) of these benefits have been a mystery.

Following the 2013 report that mTOR inhibition significantly attenuates disease in the *Ndufs4*(KO) model, great effort has been directed at defining the mechanistic underpinning of this disease attenuation. Subsequent studies have implicated multiple putative pathways, including metabolism, neurotransmitter abundance, PKC activity, redox, and transcription (5, 6, 8–11). However, no single downstream pathway has been found to reproduce the benefits of mTOR inhibition, suggesting the role of mTOR may not be defined by one discrete downstream process.

The studies detailed in this report had the goal of assessing whether the role of mTOR in the *Ndufs4*(KO) model is defined by upstream signaling, specifically, whether signaling through PI3K contributes to LS and PI3K suppression attenuates disease (for more detail, see Supplemental Figure 1; supplemental material available online with this article; https://doi.org/10.1172/jci.insight.156522DS1). Here, we report that inhibition of the PI3K catalytic subunit γ isoform p110γ/PI3Kγ, expressed primarily in leukocytes, recapitulates the benefits of mTOR inhibition, while inhibition of other PI3K isoforms does not (12). We show that directly targeting leukocyte proliferation via colony-stimulating factor 1 receptor (CSF1R) inhibition dramatically attenuates disease. CSF1R inhibition blocks CNS lesion formation, prevents neurologic signs, and rescues disease sequelae including hyperlactemia, seizures, hypoglycemia, and anesthetic responses, as well as significantly extending survival.

Note: while Iba1^+^ (ionized calcium-binding adapter molecule 1, aka allograft inflammatory factor 1, Aif1) cells are referred to as *microglia* throughout LS literature, we avoid this language for reasons detailed in the discussion section.

## Results

### Isoform-specific pharmacologic targeting of PI3Kγ significantly attenuates disease in the Ndufs4(KO) mouse.

To determine whether the benefits of mTOR inhibition in *Ndufs4*(KO) mouse result from disruption of PI3K-mediated signaling, we treated animals from weaning (postnatal day 21, P21) with potent, orally available, isoform-specific inhibitors of the catalytic subunits of PI3K: BYL719, GSK2636771, CAL-101, and IPI-549 — inhibitors of p110α, p110β, p110δ, and p110γ, respectively.

**Figure 1:**
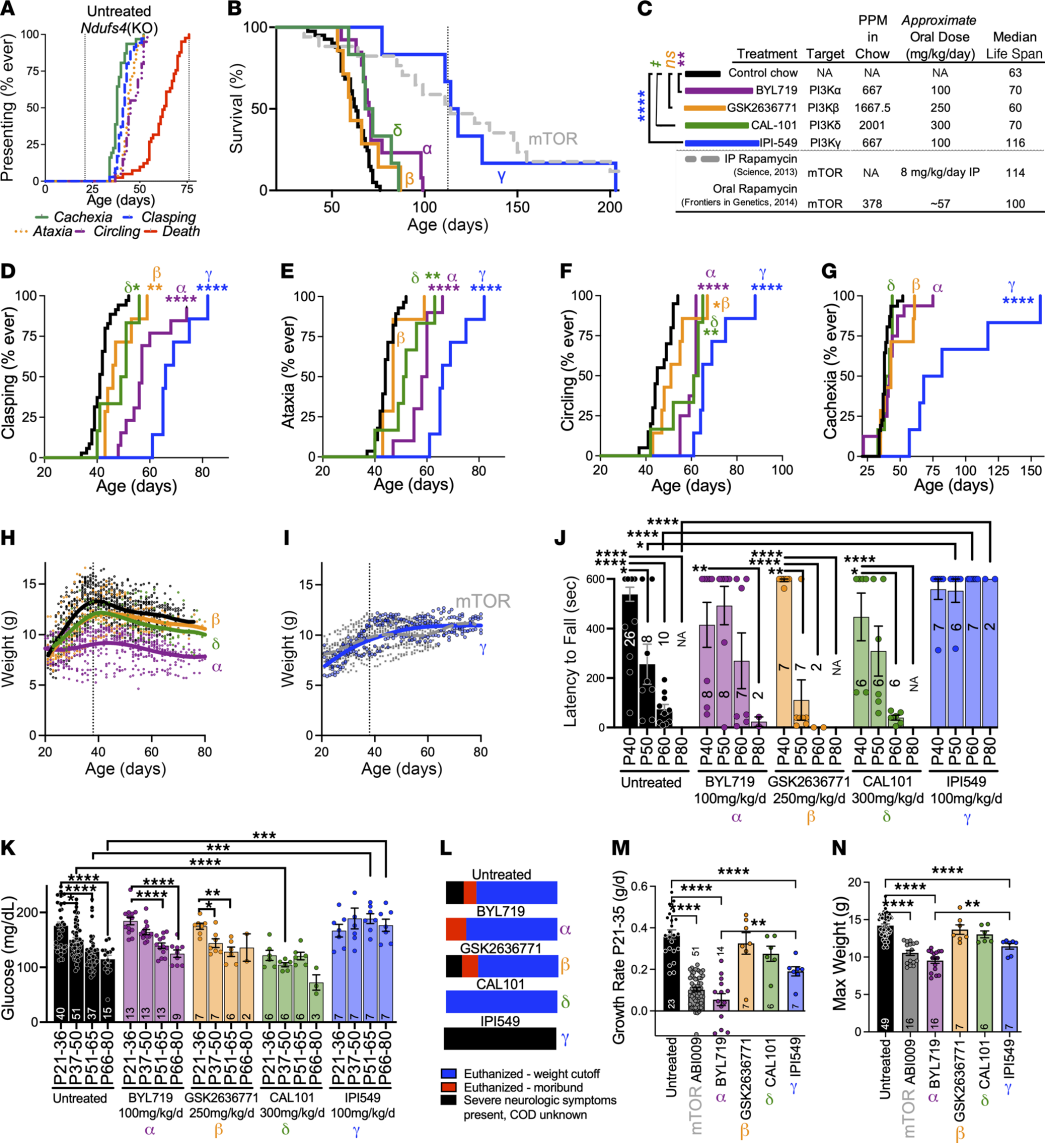
Isoform-specific inhibition of PI3K catalytic subunit p110γ, but not p110α, p110β, or p110δ, significantly attenuates disease in the *Ndufs4*(KO) mouse model of LS. (**A**) Age of cachexia (*n* = 31), clasping (*n* = 35), ataxia (*n* = 28), and circling (*n* = 20) onset and of death (*n* = 41) (see Methods for symptoms, replicates, scoring). (**B** and **C**) Survival (**B**) and life span and dosage data (**C**) for *Ndufs4*(KO) mice administered control chow (black, *n* = 41) or isoform-specific inhibitors of PI3K catalytic subunits: p110α/BYL719 (*n* = 13), p110β/GSK2636771 (*n* = 7), p110δ/CAL101 (*n* = 6), p110γ/IPI549 (*n* = 6). Rapamycin for reference (see refs. 4, 5); line, median for rapamycin. (**C**) Color key for **B** and **D**-**G**, dosing, and median life spans. ^‡^*P* = 0.017, **P < 0.005, and ****P < 0.0001, log-rank test (Bonferroni-corrected significance threshold, BCST: *P* < 0.015). (**D**-**G**) Clasping (**D**), ataxia (**E**), circling (**F**), and cachexia (**G**) in *Ndufs4*(KO) mice treated with BYL719 (*n* = 12, 10, 8, 16), GSK2636771 (*n* = 7, 7, 7, 7), CAL101 (*n* = 6, 6, 6, 6), or IPI549 (*n* = 7, 7, 7, 6). Treatment indicated by color (see **C**) and p110 α/β/δ/γ symbols. Control-treated *n*s as in **A**. *P < 0.015, **P < 0.005, ***P < 0.0005, and ****P < 0.0001 by log-rank test vs. untreated *Ndufs4*(KO) (BCST: *P* < 0.015). (**H** and **I**) *Ndufs4*(KO) weight by age and treatment (indicated by color and symbol; mTOR, ABI009 treatment); local regression (Lowess) curve overlaid. *n*s as in **B**. (**J**) Performance of *Ndufs4*(KO) mice on a rotarod assay. (**K**) Blood glucose by age (see Methods). (**J** and **K**) *n* provided as numbers within/above bars. *P < 0.015, **P < 0.005, ***P < 0.0005, and ****P < 0.0001 by Welch’s *t* test (treated vs. untreated BCST: *P* < 0.015; vs. baseline within same treatment BCST: *P* < 0.0167). For rotarod, when animals die before P80, *t* tests are interpreted as significant. (**L**) Cause of death in survival studies. *n* values as in **A** and **B**. (**M** and **N**) Growth rate P21–P35 (**M**) and maximum weight during life (**N**). *n*s indicated within bars. *P < 0.0033, ***P < 0.0005, and ****P < 0.0001, unpaired, unequal variances (Welch’s) *t* test (BCST: *P* < 0.0033). Data represent mean, error bars ± SEM, unless otherwise stated.

Control-treated *Ndufs4*(KO) animals displayed normal health early in life but rapidly developed progressive neurologic symptoms starting around P37, and death occurred by approximately P80 (Figure 1A). After around P37, animals developed progressive, degenerative lesions in the brainstem and cerebellum. Signs of neurologic decline include forelimb clasping, ataxia, and circling. Animals developed cachexia without anorexia (Supplemental Figure 2); this is the most frequent proximal cause of death, as euthanasia due to loss of body mass is the approved endpoint typically reached first (death by disease is generally not allowed in IACUC-approved animal work). The mechanistic underpinnings of this cachexia are unknown.

Treatment with the p110α, p110β, and p110δ inhibitors provided only modest (~10% or less) benefits to survival (Figure 1, B and C). In contrast, treatment with the p110γ inhibitor IPI-549 increased survival similarly to mTOR inhibition — median survival in IPI-549–treated *Ndufs4*(KO) mice was approximately 110 days versus approximately 110 and approximately 60 for rapamycin-treated and untreated animals, respectively. Drug dosing was based on published studies (13–16) (see Supplemental Methods and Discussion). Treatments led to similar tissue levels (Supplemental Figure 3).

Each of the PI3K catalytic subunit inhibitors modestly delayed at least some overt signs of neurologic disease (clasping, circling, ataxia), but IPI-549 provided the greatest benefits (Figure 1, D–G). In contrast, only IPI-549 delayed/prevented cachexia (Figure 1, G–I), and only IPI-549 improved *Ndufs4*(KO) performance on a rotarod assay, which assesses neurologic function and overall health (Figure 1J). Further, IPI-549 alone prevented progressive hypoglycemia (Figure 1K). In addition to being the only PI3K catalytic subunit inhibitor to increase survival (detailed above), IPI-549 alone qualitatively affected cause of death (Figure 1L).

mTOR inhibition with ABI-009 (nab-rapamycin, a rapamycin formulation for injection, see Supplemental Methods) at doses sufficient to attenuate disease significantly reduced developmental weight gain and maximum body size (Figure 1, I, M, and N), as we have reported previously for oral and injected rapamycin (4, 5). IPI-549 and mTOR inhibition similarly modified disease, while IPI-549 had a milder impact on growth (Figure 1M). IPI-549 also did not cause low glucose as we have observed with chronic rapamycin treatment (see ref. 17). In contrast, BYL719 severely impaired size, significantly more than IPI-549, while not rescuing disease (Figure 1, B, H, I, M, and N); these data indicate mTOR inhibition does *not* benefit LS through actions on insulin/insulin like growth factor 1 signaling, which is mediated by p110α (18).

We also tested a pan-PI3K inhibitor, BKM-120, finding that while well tolerated in adult mice at 60 mg/kg/d (19), BKM-120 was not tolerated at 50 or 100 mg/kg/d when administered from weaning (Supplemental Figure 4). Given our IPI-549 results, we did not explore this strategy further.

### mTOR inhibition reduces Iba1^+^ leukocyte proliferation in vitro.

p110γ is primarily expressed in leukocytes (Supplemental Figure 5), including the brain’s resident macrophages, microglia. Lesions in LS are characterized in part by the accumulation of Iba1^+^ cells (typically referred to as “microgliosis”; see Discussion regarding use of *leukocyte* versus *microglia*). This has widely been thought a secondary reaction to CNS cell death caused by some combination of “energetic depletion,” ROS damage, lactic acidosis, and excitotoxicity (3, 20). Given that p110γ and mTOR inhibitors provide similar benefits, we reasoned that leukocyte (including microglia) proliferation may be causal in CNS lesion formation and degeneration, rather than simply a secondary response.

**Figure 2:**
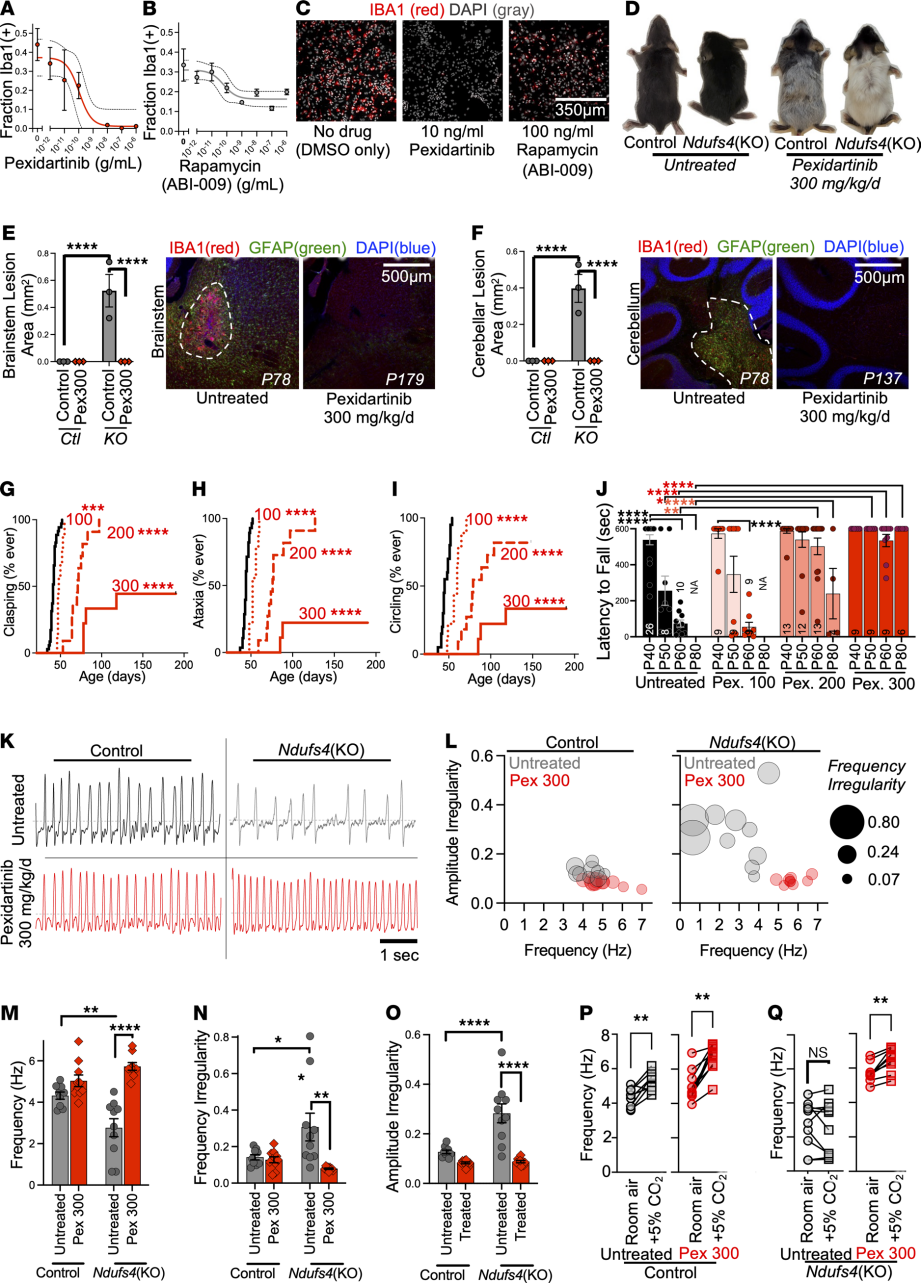
Leukocyte depletion prevents CNS lesions and significantly attenuates disease in the *Ndufs4*(KO) model of LS. (**A** and **B**) Dose-dependent impact of the CSF1R inhibitor pexidartinib (**A**) and rapamycin (ABI-009 formulation) (**B**) on the fraction of Iba1^+^ leukocytes (likely microglia given cell origins, see Methods) in mixed primary brain cultures. Error bars show ± SEM. Dashed lines, 95% confidence interval for inhibitor vs. response (3 parameters) least squares fit. *n* = 3 replicates/concentration for rapamycin, 6/condition for pexidartinib (data representative of 3 independent experiments). (**C**) Representative images of mixed primary brain cultures from **A** and **B** stained with an anti-Iba1 antibody (red) and DAPI (blue, nuclei). Scale bar in rapamycin image is representative for all. (**D**) Representative pictures of control and *Ndufs4*(KO) animals fed control diet or ~300 mg/kg/d pexidartinib chow. (**E** and **F**) Brainstem (**E**) and cerebellar peduncle (**F**) lesion size (area of lesion in central slice in serial sectioning, see Methods) in control-treated (Untreated) and 300 mg/kg/d pexidartinib (Pex300) treated control (Ctl) and *Ndufs4*(KO) (KO) animals. Quantification and representative images provided for each region. Representative images are only provided for *Ndufs4*(KO) animals as control mice do not develop lesions. See Figure 3 for quantification of Iba1^+^ leukocytes and GFAP^+^ astrocytes in control and *Ndufs4*(KO) mice. Anti-Iba1 antibody staining in red, DAPI (nuclei) in grayscale (see Methods). Lesion areas indicated by dashed white lines. Ages are noted in representative images (P78, etc.). ****P < 0.0001 by unpaired, unequal variances (Welch’s) *t* test. *n* = 3 animals per condition. (**G**–**I**) Onset of clasping (**G**), ataxia (**H**), and circling (**I**) in *Ndufs4*(KO) mice fed control diet (black lines, *n* as in Figure 1; see Methods) or administered pexidartinib at 100 mg/kg/d (dotted red lines, *n* = 9, 9, 9), 200 mg/kg/d (dashed red lines, *n* = 11, 11, 11), or 300 mg/kg/d (solid red lines, *n* = 9, 9, 9). ***P < 0.0005, and ****P < 0.0001 by log-rank test vs. untreated *Ndufs4*(KO) animals (Bonferroni significance threshold = *P* < 0.0167). (**J**) Performance of control- and pexidartinib-treated animals on a rotarod assay. **P < 0.005, and ****P < 0.0001 by unpaired, unequal variances (Welch’s) *t* test (BCST = *P < 0.0167 for both comparisons between pexidartinib-treated and control untreated mice and for comparisons to baseline within same treatment). Replicates (*n*s) are shown by vertically oriented numbers within/above bars. For rotarod, where animals do not survive to P80, *t* test undefined but interpreted as highly significant (see Methods). (**K**) Representative traces of respiratory (breathing) activity in P70 (±2 days) control and *Ndufs4*(KO) mice control treated or fed 300 mg/kg/d pexidartinib chow. (**L**) Multivariable plotting of respiratory amplitude irregularity, frequency, and frequency irregularity in P70 (±2 days) control and *Ndufs4*(KO) mice fed control chow or administered 300 mg/kg/d pexidartinib. (**M**) Frequency of breathing (representative data in **K**). (**N** and **O**) Single-variable analysis of data in **L**. (**M**–**O**) Data points represent individual animals, *P < 0.05, **P < 0.005, and ****P < 0.0005 by 2-way ANOVA with Tukey’s multiple-testing correction–adjusted *P* values for pairwise comparisons. Each comparison is pairwise. (**P** and **Q**) Respiratory responses to increased environmental CO_2_. Pairwise data shown for responses in individual mice. **P < 0.005 by Wilcoxon matched pairs signed-rank test. For all panels, data represent mean, error bars ± SEM, unless otherwise stated. (**M**–**Q**) *n* = 9 for control animal data sets, 10 for *Ndufs4*(KO) data sets.

To assess whether mTOR inhibition affects leukocyte/microglia proliferation in vitro, we tested the impact of ABI-009 (aka nab-rapamycin, see refs. 17, 21), a water-soluble nanoparticle formulation of rapamycin (see Supplemental Methods), on Iba1^+^ cells in a mixed brain cell culture assay, comparing the impact with that of pexidartinib/PLX3397, a CSF1R inhibitor that blocks leukocyte survival signaling (22) (Figure 2A). ABI-009 and pexidartinib both reduced the fraction of Iba1^+^ cells in mixed neonatal brain cell cultures in a dose-dependent manner. The maximum effects of ABI-009 and pexidartinib were about 50% and about 100% depletions, respectively. Accordingly, mTOR inhibition does appear to preferentially limit leukocyte proliferation compared with other cell types, but the potency is limited compared with directly targeting leukocyte survival.

### Leukocyte depletion prevents CNS lesions and neurologic sequelae.

Taken together, our data suggested that leukocyte proliferation may causally drive disease in LS.

To test this model, we treated *Ndufs4*(KO) and control animals with 100, 200, or 300 mg/kg/d pexidartinib in normal mouse chow (dosing approximated based on food consumption, see Supplemental Methods; tissue drug levels in Supplemental Figure 6; note — higher doses led to a change in mouse coat color, consistent with reports of hair whitening in humans; Figure 2).

Treatment with 300 mg/kg/d pexidartinib led to a complete (by IHC) prevention of brainstem and cerebellar (Figure 2) lesions in *Ndufs4*(KO) mice, even at ages far beyond the maximum survival of untreated animals. Critically, both accumulation of Iba1^+^ cells *and* astrocytosis were completely prevented by pexidartinib, indicating that astrocyte involvement is secondary to leukocyte activity.

Consistent with these findings, pexidartinib dose dependently delayed the onset and reduced the incidence of behavioral signs of neurodegeneration — forelimb clasping, ataxia, and circling (Figure 2, G–I). Pexidartinib treatment also rescued performance on the rotarod assay (Figure 2J).

Impaired respiration, which is a brainstem function, is a proximal cause of death in patients with LS and has been reported to be a proximal cause of death in *Ndufs4*(KO) mice not euthanized (23). We performed plethysmography in untreated and 300 mg/kg/d pexidartinib–treated mice to assess respiratory function (see ref. 24 for diagram and Supplemental Methods for details). Severe defects in respiratory function were present in untreated *Ndufs4*(KO) mice at about P70 in respiratory frequency, incidence of amplitude and frequency irregularities, and responses to increased CO_2_ (Figure 2). Treatment with 300 mg/kg/d pexidartinib completely prevented each of these respiratory defects (Figure 2).

### Leukocyte depletion rescues neuroinflammation outside of lesions.

**Figure 3:**
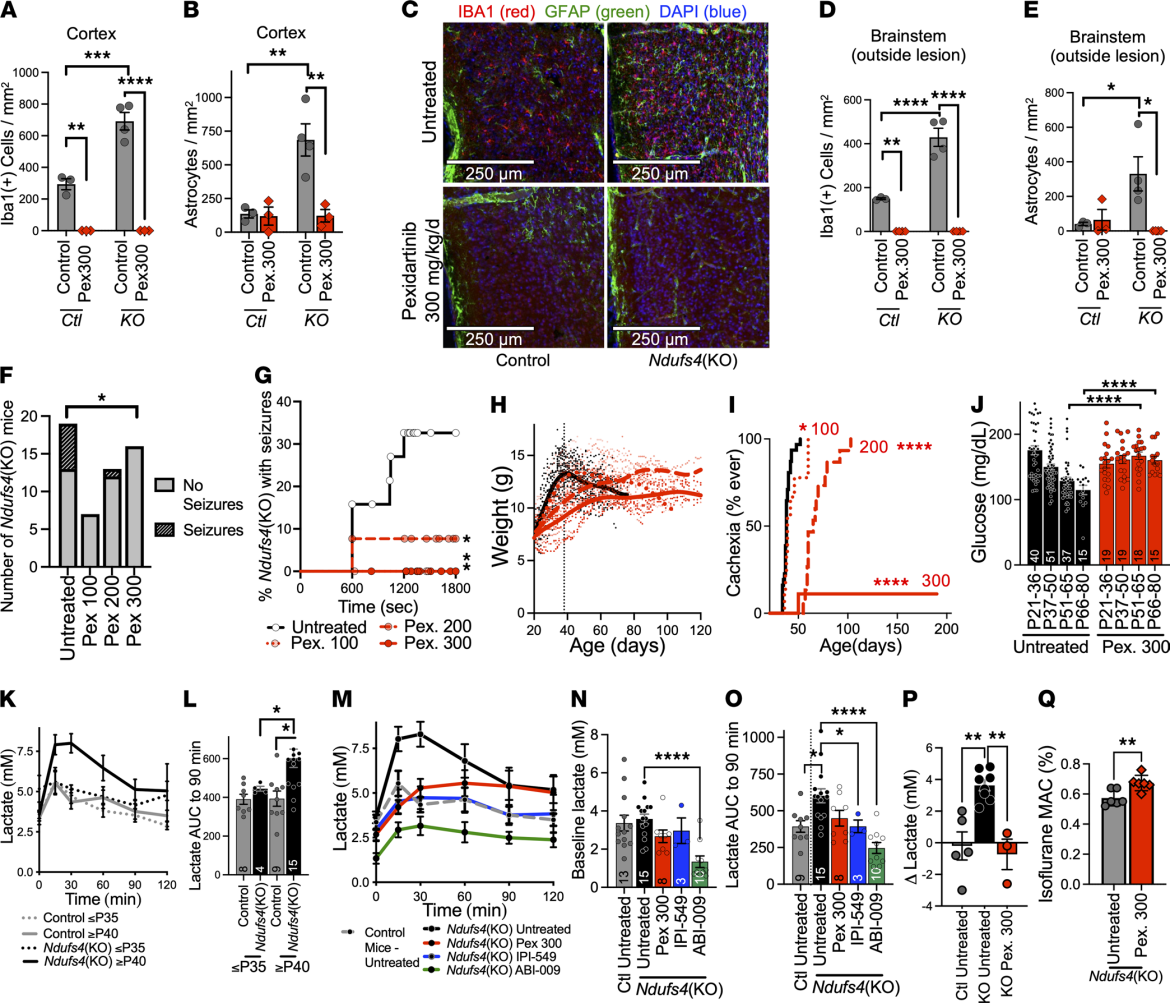
Leukocyte depletion prevents leukocyte/microglia accumulation and astrocytosis throughout the brain and rescues a range of systemic symptoms associated with LS in the *Ndufs4*(KO) mice. (**A**–**C**) Iba1^+^ leukocytes/microglia (**A**) (see Discussion) and GFAP^+^ astrocytes (**B**) in cortex of control- and pexidartinib-treated control and *Ndufs4*(KO) (see Methods). Data points represent individual animals. *n* = 4 for untreated *Ndufs4*(KO), 3 for other groups. (**C**) Representative images of cortex. (**D** and **E**) Iba1^+^ leukocytes/microglia and GFAP^+^ astrocytes in brainstem regions outside overt lesions in control- and pexidartinib-treated control and *Ndufs4*(KO) mice (representative images in Figure 2). Data points represent individual animals. *n* = 4 for untreated *Ndufs4*(KO), 3 for other groups. *P < 0.05, **P < 0.005, ***P < 0.0005, and ****P < 0.0001, 2-way ANOVA with Tukey’s multiple-testing correction–adjusted *P* values for pairwise comparisons. (**F**) Rotarod-induced seizure frequency at P30 by treatment. *P < 0.016, Fisher’s exact test (Bonferroni-adjusted *P* value cutoff for significance = 0.0167). *n* indicated by bars. (**G**) Time to seizure in rotarod assay, P30. All data points shown. *P < 0.05, log-rank test. *n*s as in **F**. (**H**) Scatter plots of *Ndufs4*(KO) mouse weight as a function of age and treatment, with local regression (Lowess) curves to display population trends. (**I**) Cachexia onset (see Figure 1, Methods) in control- and pexidartinib-treated *Ndufs4*(KO) mice. Colors and *n*s as in **G**. *P < 0.0167, ****P < 0.00005, log-rank test (Bonferroni-corrected *P* value cutoff for significance = 0.0167). (**J**) Blood glucose by age in control- and pexidartinib-treated *Ndufs4*(KO) animals. Each point represents the median value for 1 animal during the period (data points are biological replicates/individual animals). ****P < 0.0001 by unpaired, unequal variances (Welch’s) *t* test (Bonferroni-corrected *P* value cutoff for significance = 0.0125). *n*s as indicated in bars. (**K** and **L**) Blood lactate in response to a glucose bolus (2 g/kg) in control and *Ndufs4*(KO) mice at predisease (P25) and early disease (P45). (**K**) Time course and (**L**) total AUC for blood lactate 0–90 minutes. *P < 0.008, 2-way ANOVA with Tukey’s multiple-testing correction–adjusted *P* values for pairwise comparisons. *n*s indicated in bars. (**M**–**O**) Blood lactate in response to glucose bolus (2 g/kg) in untreated control and *Ndufs4*(KO) mice and *Ndufs4*(KO) mice treated with pexidartinib (300 mg/kg/d in chow), IPI-549 (100 mg/kg/d in chow), or rapamycin (ABI-009 formulation, 8 mg/kg/d IP). Time course of blood lactate (**M**), baseline lactate (**N**), and total AUC for blood lactate (**O**) 0–90 minutes. *P < 0.0167, **P < 0.005, ****P < 0.0001 by unpaired, unequal variances (Welch’s) *t* test (Bonferroni-adjusted significance cutoff = 0.0167). *n*s indicated in bars (**N** and **O**). (**P**) Change in blood lactate in control and *Ndufs4*(KO) mice after 30-minute exposure to 0.4% isoflurane and impact of treatment with 300 mg/kg/d pexidartinib. *n* = 5, 7, and 3. **P < 0.005 by 1-way ANOVA with Tukey’s multiple-testing correction–adjusted *P* values for pairwise comparisons. (**K** and **M**) AUC, not individual time points, were compared. (**Q**) Minimum alveolar anesthetic concentration (MAC) of isoflurane associated with anesthesia in control- and pexidartinib-treated *Ndufs4*(KO) mice (see Methods). **P < 0.005 by unpaired, unequal variances (Welch’s) *t* test. *n* = 6/group. Data represent mean, error bars ± SEM, unless otherwise stated.

Overt lesions in the brainstem and cerebellum underlie many features of LS, while inflammation in other CNS regions has not been carefully studied. Given the robust impact of pexidartinib in preventing lesions, we wondered whether neuroinflammation is present, and responsive to pexidartinib, in other brain regions. To assess this possibility, we analyzed Iba1^+^ and GFAP^+^ (astrocyte) cell numbers in cortex and in brainstem tissue outside overt lesions. Untreated *Ndufs4*(KO) mice showed increases in Iba1^+^ cells and astrocytes in nonlesion CNS tissue, while 300 mg/kg/d pexidartinib prevented both markers of neuroinflammation (Figure 3, A–E; see also Supplemental Figure 7). Notably, pexidartinib treatment led to a near-complete depletion of Iba1^+^ cells while astrocytes were rescued to control levels, again indicating that astrocytosis is secondary to leukocyte involvement. As with lesions, the benefits of pexidartinib extended to ages far beyond the maximum life span of untreated *Ndufs4*(KO) mice (see Figure 3 legend for details).

### Pexidartinib treatment prevents rotarod-induced seizures.

Given the inflammation in multiple CNS regions, we next wondered if LS features not previously linked to the lesions, such as seizures, might also be rescued by targeting leukocytes. Epileptic seizures are common in LS and often refractory to standard therapies (25).

The rotarod assay provided a mild epileptogenic stimulus in the *Ndufs4*(KO) model: seizures occurred in approximately 30% of untreated *Ndufs4*(KO) mice, but were not observed in control animals, during rotarod at age P30 (17) (Figure 3F and Supplemental Methods). We performed rotorod assay on control and pexidartinib-treated *Ndufs4*(KO) mice to assess whether targeting leukocytes impacts seizures. Incidence and time to seizure were both significantly reduced by pexidartinib (Figure 3, F and G).

### Hyperlactemia is prevented by rapamycin, IPI-549, and pexidartinib.

Metabolic defects are major sequelae of GMDs. As noted, treatment with pexidartinib prevented both cachexia and hypoglycemia in the *Ndufs4*(KO) mice in a dose-dependent manner (Figure 3, H–J) (5), consistent with leukocytes playing a role in metabolic derangements. We next considered the possibility that leukocyte activity may drive other metabolic sequelae in LS.

Abnormally high blood or CNS lactate (often assessed by lactate/pyruvate ratio) is frequently reported in LS and some other forms of GMD (26–29). In addition, increased lactate is a feature of LS CNS lesions when imaged by magnetic resonance spectroscopy (MRS), and increased intracerebral lactate by MRS has been reported in *Ndufs4*(KO) mice (30–32). The *cellular* origins of increased lactate in GMD have not been defined. Leukocytes are highly glycolytic (33), so a role for leukocytes in contributing to lactate appeared reasonable.

To probe hyperlactemia in untreated *Ndufs4*(KO) mice, we measured blood lactate at baseline and in response to a glucose bolus in a glucose tolerance test (GTT) paradigm. Clearance of glucose was not significantly altered in the *Ndufs4*(KO) animals, and blood lactate was not increased in *Ndufs4*(KO) animals at baseline; however, exposure of *Ndufs4*(KO) animals to a glucose bolus resulted in a significant rise in blood lactate not observed in control mice (Figure 3, K and L, and Supplemental Figure 8). Notably, this glucose-induced hyperlactemia occurred only in *Ndufs4*(KO) mice older than P37, when CNS symptoms present. Pexidartinib treatment prevented the glucose-induced hyperlactemia, supporting the leukocyte model.

Given the clinical importance of hyperlactemia, we next tested the impact of mTOR or PI3Kγ inhibition on GTT-induced hyperlactemia, finding that both ABI-009 and IPI-549 prevented hyperlactemia in response to a glucose bolus (Figure 3, M–O). Only rapamycin/ABI-009 significantly reduced baseline lactate.

### Pexidartinib attenuates anesthetic sensitivities in the Ndufs4(KO) model.

Hypersensitivity to both sedation and toxicity from volatile anesthetics is seen in mitochondrial mutants from invertebrates to mammals, including *Ndufs4*(KO) mice and human patients with electron transport chain complex I (ETC CI) defects (34, 35). Mechanisms are poorly understood, but off-target effects include metabolic disruption; low-dose isoflurane exposure leads to a blood lactate spike in *Ndufs4*(KO) mice but not control animals (Figure 3P).

Given the impact of pexidartinib on lactate during the GTT, we tested whether pexidartinib affects isoflurane-induced hyperlactemia. Treatment fully suppressed this lactate spike in *Ndufs4*(KO) animals (Figure 3P).

To test whether pexidartinib affects hypersensitivity to sedation, we measured the tail-clamp MAC (see ref. 36) of isoflurane in control and 300 mg/kg/d pexidartinib–treated *Ndufs4*(KO) mice (see Supplemental Methods for details). We tested P30 animals to assess sensitivity in the absence of overt neurologic disease. Pexidartinib modestly but significantly attenuated hypersensitivity in the *Ndufs4*(KO) mice (Figure 3Q).

### Pexidartinib increases Ndufs4(KO) life span, while drug toxicity limits survival.

**Figure 4:**
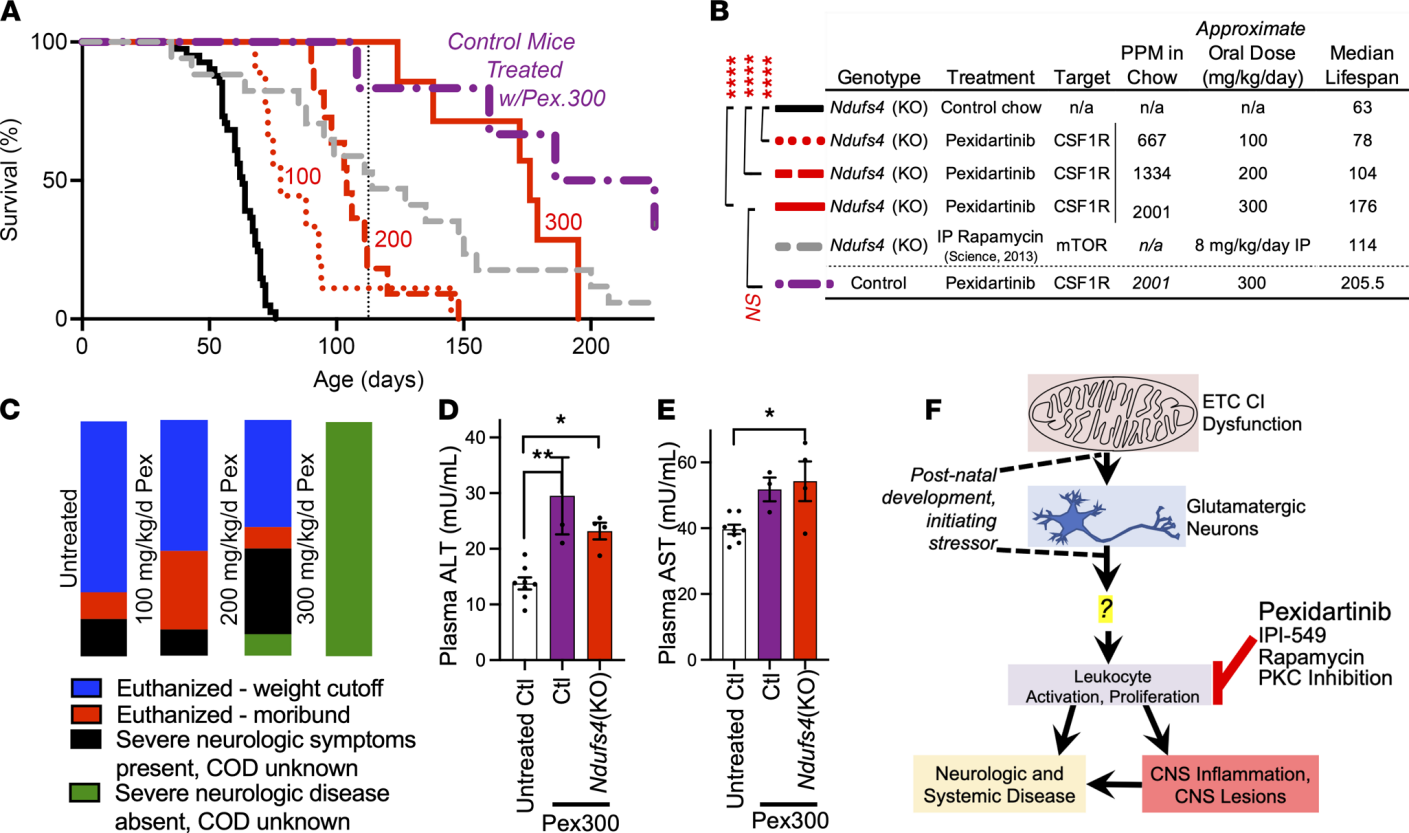
Pexidartinib dose dependently increases *Ndufs4*(KO) survival, with survival limited by drug toxicity rather than CNS disease. (**A** and **B**) Survival and cause of death in *Ndufs4*(KO) mice treated with increasing doses of pexidartinib. (**A**) Survival curves. Black line — control-treated *Ndufs4*(KO). Red dotted, dashed, and solid lines — *Ndufs4*(KO) mice treated with 100, 200, or 300 mg/kg/d pexidartinib, respectively (*n* as in Figure 2, G–I). Purple dashed/dotted line — control animals treated with 300 mg/kg/d pexidartinib (*n* = 6). Gray dashed line — rapamycin life span (for reference, see Figure 1). (**B**) Median life spans and dosing data associated with **A**. ****P < 0.0001 by log-rank test (passing Bonferroni’s cutoff of *P* < 0.0167). (**C**) Cause of death for *Ndufs4*(KO) animals in control and pexidartinib treatment groups (all control animals on pexidartinib 300 mg/kg/d died of unknown causes with no overt signs of disease/illness). *n* as in **A**. (**D**) Plasma ALT and (**E**) plasma AST levels (see Supplemental Methods). Data represent mean, error bars ± SEM. *P < 0.05, **P < 0.005 by 1-way ANOVA with Tukey’s multiple-testing correction–adjusted *P* values for pairwise comparisons. *n* = 8, 3, and 4 for untreated controls and pexidartinib 300 mg/kg/d treated controls and *Ndufs4*(KO) animals, respectively. (**F**) A model for the pathogenesis of disease in LS (see Discussion). In this model, CNS lesions and many systemic sequelae are causally downstream of immune involvement. A central role for glutamatergic neurons in initiating disease has previously been identified (8, 40), while our findings here reveal a role for leukocytes in the pathogenesis of disease. In the model resulting from these combined data, the mechanisms underlying the benefits of rapamycin and of PKC inhibitors, previously shown to benefit the *Ndufs4*(KO) mouse, can be accounted for by their convergence with pexidartinib and IPI-549 on leukocyte suppression. Key remaining questions are presented in Discussion.

Pexidartinib extended survival of *Ndufs4*(KO) animals in a dose-dependent manner (Figure 4, A and B). Critically, the survival curves of control and *Ndufs4*(KO) animals treated with 300 mg/kg/d pexidartinib overlapped, indicating that the treatment, rather than the underlying disease, is limiting survival at this dose. Consistent with this notion, the majority of high-dose pexidartinib animals did not show overt signs of neurologic sequelae prior to death; the proximal cause of death was unclear for both *Ndufs4*(KO) and control animals at this dose (Figure 4C; see Discussion).

Chronic pexidartinib is associated with risk of serious cholestatic or mixed liver injury in human patients; hepatic function is carefully monitored in patients taking this drug (37). We tested blood alanine aminotransferase (ALT) and aspartate aminotransferase (AST), established blood markers for pexidartinib-induced hepatic damage (38, 39), in treated and untreated mice to determine whether hepatotoxicity may contribute to early mortality. Consistent with this possibility, pexidartinib-treated control and *Ndufs4*(KO) mice at approximately P150 showed significantly elevated ALT and AST compared with approximately P150 untreated controls [note: *Ndufs4*(KO) mice perished prior to this age] (Figure 4, D and E).

### Inflammatory chemokines are increased in Ndufs4(KO) brainstem at ages associated with disease.

Finally, a cursory analysis of chemokines in brainstem supports both immune involvement and a post-P37 onset (detailed in Supplemental Figure 9). Among a targeted set of chemokines, 4 showed significantly altered expression in *Ndufs4*(KO) mice compared with controls. At P25, before onset of overt disease, only the eosinophil-associated molecule Eotaxin was significantly elevated. At P45 IFN-γ, IFN-γ–induced protein 10 (IP-10/CXCL10), and leukemia inhibitory factor were all increased in *Ndufs4*(KO) mice, while VEGF was reduced. Detailed exploration of immune involvement in LS is beyond the scope of this manuscript, but this analysis identifies putative chemokine mediators that should be of significant interest for follow-up study.

### A model for the pathogenesis of Leigh syndrome.

Symptoms develop in the *Ndufs4*(KO) postnatally (see Figure 1), reminiscent of human patients, who are often healthy at birth. Neuron-specific (Nestin-Cre) or glutamatergic neuron-specific (VGlut2-Cre) deletion of *Ndufs4* recapitulates disease, including CNS lesions, cachexia, metabolic dysfunction, neuroinflammation, and reduced survival, while *Ndufs4* deletion in other neuron types does not (8, 40, 41). Considering these data, we can assemble a model for the pathogenesis of disease in the *Ndufs4*(KO): mitochondrial dysfunction interacts with development to precipitate cellular sequelae in VGlut2^+^ neurons. Glutamatergic neuron dysfunction leads to immune activation and leukocyte recruitment/proliferation with chemokine induction. Leukocyte accumulation is subsequently a causal force in CNS lesions, astrocytosis, CNS dysfunction, and many systemic symptoms (see Figure 4). The final sentence is the key finding of this study.

## Discussion

### A causal role for leukocyte proliferation in the pathobiology of LS.

Our key findings are that leukocytes are a causal cellular mediator of the pathogenesis of LS and that targeting leukocytes prevents CNS lesions and substantially mitigates disease. Simply put, we have provided evidence that the immune system mechanistically drives pathology in this model of LS, necessitating a reconsideration of current models for the pathobiology of the disease.

Leukocyte depletion not only prevented sequelae associated with overt CNS lesions (e.g., respiratory dysfunction, balance, survival) but also sequelae not attributed to the CNS lesions — hypoglycemia, cachexia, hyperlactemia in response to glucose or anesthesia, seizures, and so on. Whether this indicates these symptoms are downstream of CNS involvement, or peripheral leukocyte activity drives symptoms, remains to be determined.

The rescue of glucose- and isoflurane-induced hyperlactemia was, perhaps, the most unexpected benefit of pexidartinib. This finding indicates that leukocytes mediate increased blood lactate either directly (via leukocyte cellular metabolism) or indirectly, via the consequences of inflammation (e.g., tissue ischemia or metaboregulatory consequences of CNS degeneration). Distinguishing these possibilities will be important in future studies. Additionally, the lactate findings may prove relevant to other forms of GMD involving hyperlactemia.

### Evidence for peripheral leukocytes in LS CNS lesions.

While microglia are a likely suspect in driving CNS pathologies, we present our findings in terms of leukocytes as a broad category; Iba1, rapamycin, IPI-549, and pexidartinib are not specific to microglia, and our data do not distinguish between microglia and peripheral leukocytes.

LS lesions have been defined by “microgliosis” since the initial description of the disease by Denis Leigh in 1951, but it is critical to note that this designation was based on Nissl staining, which is nonspecific (42). Iba1 staining is, unfortunately, not microglia specific — Iba1 is expressed in all macrophages and has recently been termed a pan-macrophage marker (43, 44) — but studies in *Ndufs4*(KO) animals have relied on Iba1 staining to identify “microgliosis” without ever noting the lack of microglia specificity (41). Accordingly, use of *microgliosis* in describing LS lesions is misleading — evidence does support the presence of microglia but does not rule out involvement of nonmicroglial Iba1^+^ cells.

A detailed investigation of the immune component of LS lesions is a critical next step but is beyond the scope of this manuscript. However, preliminary evidence indicates that peripheral leukocytes contribute *significantly* to LS lesions: brains of *Ndufs4*(KO) mice expressing TMEM119-GFP (a microglia-specific marker, refs. 45, 46) costained for GFP and Iba1 revealed that a substantial portion of cells within lesions are Iba1^+^ but TMEM119^–^ (Supplemental Figure 10 and Supplemental Methods). These data reveal that peripheral immune cells are important contributors to LS lesions. Defining the cellular composition of LS CNS lesions may be critically important to understanding this disease.

### A mechanism for mTOR inhibition in LS.

Determining the mechanism underlying the benefits of rapamycin in the *Ndufs4*(KO) model has been an active area of research since the 2013 study. Processes including metabolism, neurotransmitter abundance, PKC activity, and regulation of transcription have been implicated, but no single downstream pathway has recapitulated the benefits of mTOR suppression (4–6, 8–11, 47–52). Our data support a model whereby these benefits result *primarily* from immune modulation. Critically, it does not appear any of the prior studies on mTOR in *Ndufs4*(KO) animals are incompatible with our findings; some even hinted at immune involvement (for example, the PKC study authors note that PKC inhibitors play a role in mTOR-mediated immune regulation, ref. 11).

It is important to note that mTOR inhibitors have beneficial effects in a variety of GMD models, including cultured cells and invertebrates (6, 48–52). mTOR inhibition is pleiotropic, and other mTOR-regulated processes may mediate the benefits of targeting mTOR in other settings. On the other hand, immune activation may play a previously unappreciated role in forms of GMD other than LS, a possibility warranting further attention.

We cannot rule out the possibility that p110α, p110β, and p110δ play some minor role in disease. Alternative targeting methods/agents might be more effective. Conversely, the modest benefits observed with the p110α, p110β, and p110δ inhibitors might simply result from off-target effects on p110γ. Targeted manipulation of mTOR, p110γ, or CSF1R in leukocytes and/or microglia may provide a more potent benefit with reduced off-target effects, and such experiments might provide further clarity to the pharmacologic results.

### Therapeutic implications.

Our findings may have significant therapeutic implications. Clinical translation is exceptionally difficult in rare diseases (53–56), compounded in LS by postnatal onset, difficulties in diagnosis, and clinical and genetic heterogeneity (>75 causal genes) (57–59). mTOR inhibition was the first pharmacologic agent shown to attenuate disease in the *Ndufs4*(KO) model and benefits at least some patients (6, 7, 48). However, the translational potential of mTOR inhibitors is limited by incomplete rescue and well-documented off-target effects, among other issues.

We do not suggest that the specific compounds used here will, or should, be trialed in patients. It is the discovery that leukocytes mediate disease which opens the door for an entirely new realm of therapeutic targets. Many well-characterized, clinically approved agents already exist for targeting immune function. We utilized CSF1R inhibition to deplete leukocytes, but this strategy was an experimental tool — untargeted and extreme. Follow-up studies are likely to identify novel agents targeting immune activation in LS with improved potency and reduced off-target effects.

Our findings may apply to other GMDs where inflammation has been observed. Determining the generalizability of our findings to genetically distinct forms of LS and other GMDs will be an important goal of future studies.

### New insights, new questions.

Our data appear to resolve some mysteries that have persisted in the study of LS. In particular: 1) Why is there a general sparing of some tissues with high energy requirements, as in cardiac-specific *Ndufs4*(KO) in the absence of a stressor (60–62)? If disease sequelae are mediated by immune responses, tissue energetics may not play a *direct* role. 2) Why does LS typically develop postnatally, without symptoms at birth? Viral infection and fever have been reported to often coincide with symptom onset in LS and other forms of acute focal necrotizing encephalopathy in children (63–69), a link attributed by some to “energetic stress” induced by mobilizing an immune response (65). Our data might indicate that immune induction directly contributes to this link, rather than by precipitating an energetic crisis.

While we believe our data represent a significant step forward in our understanding of LS, and perhaps GMD broadly, key questions remain. What *precise* VGlut2^+^ cells initiate inflammation? Do any non-CNS sequelae result from peripheral inflammation? What role, if any, do leukocytes play in other forms of GMD? Is there a link between inflammation and chronic hypoxia, the most potent therapeutic strategy yet identified in the *Ndufs4*(KO) model (70)? Finally, how does altered mitochondrial function drive immune activation? One might hypothesize that mitochondrial dysfunction leads to sensing of some mitochondrial component, perhaps mtDNA or misfolded proteins, as “foreign” by the immune machinery. The cyclic GMP-AMP synthase/stimulator of IFN genes pathway, which senses intracellular DNA and induces type 1 IFN, provides one candidate for further study but will require identification/isolation of the initiating cell type(s) (71, 72). If an immune-activating factor, or factors, can be identified, they may provide additional insight into the curious postnatal onset and unique CNS involvement in LS.

Answers to these questions will shed new light on the interplay between mitochondria, the immune system, and disease. Future studies utilizing targeted genetic manipulation of key immune components will be particularly important in clearly defining the role of the immune system in LS.

## Methods

### Animal sources and protocol.

*Ndufs4*^+/–^ mice (originally obtained from the Palmiter laboratory, University of Washington, Seattle, Washington, USA; available through The Jackson Laboratory, strain 027058) were bred to produce *Ndufs4*(KO) (*Ndufs4*^–/–^) offspring. Mice were weaned at 20–21 days of age. KO mice were housed with control littermates for warmth and stimulation. Mice were weighed and health was assessed a minimum of 3 times per week (daily for i.p.-injected mice; see below). Where longitudinal blood point-of-care data were collected, this was performed during health checks. Wetted chow in a dish on the bottom of each cage, and in-cage water bottles, were provided to cages housing *Ndufs4*(KO) mice following onset of symptoms so that food and water accessibility was never a limiting factor for survival.

Staining in Supplemental Figure 9 used an *Ndufs4*(KO) mouse expressing TMEM119-GFP (carrying 1 copy). This reporter line was obtained from The Jackson Laboratory (strain 031823, C57BL/6 strain) and bred into the *Ndufs4*(KO) line.

Mice were euthanized if they reached a 20% loss in maximum body weight or were immobile or found prostrate or moribund.

As previously reported, *Ndufs4* deletion is a recessive defect, and heterozygosity results in no reported phenotypes and no detectable defects in ETC CI activity; controls for this data set included both heterozygous and WT mice.

All experimental mice were fed PicoLab Diet 5058; pharmacologic agents were compounded into this diet (see Supplemental Methods).

### Study approval.

Animal experiments followed Seattle Children’s Research Institute guidelines, and experiments were performed as approved by the IACUC of Seattle Children’s Research Institute.

## Author contributions

SCJ conceived the study. SCJ, JC Stokes, RLB, KJ, NAB, KYP, and KAS designed the methodology. SCJ, JC Stokes, RLB, KJ, NB, KYP, KAS, BMJ, JC Snell, and KV investigated. SCJ, RLB, and NAB visualized data. SCJ and MS acquired funding. SCJ performed project administration. SCJ, MMS, and PGM supervised. SCJ wrote the original draft. SCJ, MMS, PGM, and RLB reviewed and edited the draft. For authorship order among co–first authors: order was assigned based on relative contributions and was agreed on by all co–first authors.

## Supplementary Material

Supplemental data

## Version 1 01/20/2022

In-Press Preview

## Version 2 03/08/2022

Electronic publication
