# Supplementary material for: Leukocytes mediate disease pathogenesis in the Ndufs4(KO) mouse model of Leigh syndrome
Source: JCI Insight. 2022 Mar 8;7(5):e156522. doi: 10.1172/jci.insight.156522 (PMC8983133; doi:10.1172/jci.insight.156522)
Supplement: Supplemental data [file jciinsight-7-156522-s026.pdf]

Leukocytes mediate disease pathogenesis in the *Ndufs4*(KO) mouse model of Leigh syndrome

Supplementary Materials

Supplementary Materials and Methods

Longitudinal assessments of disease

Clasping, ataxia, and circling were assessed by visual scoring, as previously described (1, 2). As disease progresses in *Ndufs4*(KO)'s animals display intermittent/transient improvement of symptoms. Here, we simply report whether animals *ever* presented the symptom for two or more consecutive days (this criterion to minimize spurious reporting). For observational assessments, lab-wide quality control discussions occurred frequently to ensure consistency between technicians/researchers. Technicians contributed equally to each treatment group to minimize any potential bias between individuals.

Cachexia onset (**Figure 1** and **Figure 3**) is simply the day of life when an individual animal's weight peaks, when progressive weight loss starts.

Reagents

Laboratory chemicals were purchased from Sigma. Pexidartinib, IPI-549, CAL-101, and GSK2636771 were purchased from MedKoo Biosciences (cat#'s 206178, 206618, 200586, and 205844, respectively). BYL719 was purchased from AChemBlock (cat#R16000).

Doses of each drug was based on published data for use in animals. Tissue concentrations reached biologically relevant levels for each of the tested inhibitors, see **Figures S2, S5**.

Pharmacologic interventions

Mouse chow was ground to powder and mixed with drugs (see table in **Figure 1**). 300mL of 1% agar melted in sterile water was added per kilogram powdered chow and the mixture was pelleted and incubated at 37°C for 3-5 hours until dry. Pellets were stored at 4°C short term (up to 30-days) or -20°C long-term (up to 6-months). We previously demonstrated that this processing has no impact on animal health, survival, or disease (1). Food consumption calculated based on data in **Figure S2** – the value of (0.15g-food/g-mouse/day), the lower end of daily food consumption estimate, was used to ensure mice received adequate drug.

Replicate numbers, controls, and ABI-009

Animal numbers for each dataset are in figure legends. Whenever possible all datapoints are shown.

Assignment to control treatment groups was spread throughout the duration of these studies to ensure that no shifts in colony survival, behavior, etc, occurred during the course of these experiments. Accordingly, control treatment groups generally contain larger cohorts than the individual treatments.

Rapamycin was provided as ABI-009 (nab-rapamycin), an albumin encapsulated water-soluble formulation. ABI-009 was provided by Aadi LLC (17383 Sunset Blvd, Pacific Palisades, CA 90272) in lyophilized form. ABI-009 was resuspended to 1.2mg/mL rapamycin in 1XPBS. This solution was sterile-filtered and stored in aliquots at -80ºC. ABI-009 was administered at 66µL/10g for a dose of 8mg/kg/day rapamycin, as in prior studies (1, 2).

Rapamycin/ABI-009 treatment groups were included when data had not been previously published. Where survival/long term treatment experiments for rapamycin/ABI-009 have already been published by our group we have provided reference to prior datasets (1-5). Critically, we do not make statistical comparisons to prior/historic data, they are provided only for the sake of immediate reference/context and are labeled as 'historic' data in each case. Omitting the historic data would have no impact on our findings or conclusions, but we feel the added context is valuable.

Point-of-care glucose and lactate testing

Blood glucose and lactate were measured using point-of-care meters (Prodigy Autocode glucose meter, cat#51850-3466188; Nova Biomedical lactate meter, cat#40828) and the tail-prick method, as previously described (6).

Rotarod and rotarod seizures

Rotarod was performed using a Med Associates ENV-571M single-lane rotarod. Assays were performed by placing animals onto an already rotating rod and timing latency to fall. Rotation was set to constant 6rpm (Med Associates software). Maximum time of each trial was 10min. For each assay, three trials were performed, with a minimum of 5min between assays. The best of the three trials was reported.

Mice were monitored during each trial for seizure activity. Seizures were identified by any of the symptoms on the Racine behavioral scale or Pinel and Rovner scales, but abnormal oroalimentary movements (dropping of the jaw repeatedly, atypical gnawing or chewing movements), and repeat head nodding were not considered 'definitive' for seizure activity and did not lead to trial halt or seizure scoring. Anterior limb clonus (twitching/jumping), dorsal extension/rearing, loss of balance and violent falling (observed when mouse was not on the rotarod), violently running/jumping were considered definitive of seizures and resulted in both a halt to trials that day and recording of a seizure incident. No attempt was made to further score seizure severity for these studies.

Glucose tolerance test, glucose induced lactate test

Prior to the GTT,mice were fasted for 4 hours, from ~10AM-2PM. Fasting was limited to 4 hours due to the sensitivity of untreated *Ndufs4*(KO) animals to hypoglycemia and related sequelae (dysregulation of body temperature, hypoglycemia induced seizure, etc). For the ABI-009 cohort, mice received their daily ABI-009 injection at the start of the fast. At the end of the fast, baseline blood glucose and lactate were measured using point-of-care meters and the tail-prick method (above). Mice were then injected by IP with 2g/kg dextrose (10 microliters/gram body weight of sterile filtered 200mg/mL glucose in 1XPBS) using an insulin syringe (31-gauge, 6mm length, 3/10cc, BD Veo Ultra-Fine). Glucose and lactate were measured using tail-prick at designated timepoints post-injection.

Isoflurane induced hyperlactemia assays

Mice were subject to normal daily monitoring and treatment (above) until P50. At P50, blood glucose, lactate, and β-HB levels were measured using point-of-care meters using the tail-prick method, as above, immediately before and after a 30-minute exposure to 0.4% isoflurane or carrier gas only (100% oxygen).

Anesthesia sensitivity

The tail-clamp minimum alveolar anesthetic concentration (MAC) was measured as previously described (7) in P30 mice. Briefly, mice were placed in individual gas-tight plastic chambers connected to an isoflurane vaporizer (Summit Anesthesia) anesthetic source set to flowrate 1-2 liters/min, humidified in-line. Mice were kept warm with a circulating water pad at 38°C (Adroit Medical, HTP-1500). Anesthetic concentration was adjusted in steps of 0.2%, equilibrated for 10-minutes per step, and monitored with an in-line analyzer (AA-8000 Anesthetic Agent Analyzer, BC

Biomedical). MAC was calculated as the mean of concentrations bracketing the animal's response and non-response to a nondamaging tail clamp.

Respiratory function

Breathing parameters were recorded from alert, unrestrained, ~P70 (+/- 2 days) mice using whole-body plethysmography. Paired 300 ml recording and reference chambers were continuously ventilated (150ml/min) with either normal air (79%N_2/21%O_2) or a hypercapnic gas mixture (74%N_2/21%O_2/5%CO_2). Pressure differences between the recording and reference chambers were measured (Buxco) and digitized (Axon Instruments) to visualize respiratory pattern, and simultaneous video recordings were performed to differentiate resting breathing activity from exploratory and grooming behaviors. Mice were allowed to acclimate to the chambers for 30-40min prior to acquisition of 35min of respiratory activity in normal air. Respiratory response to hypercapnia was then tested by ventilating the chambers with hypercapnic gas for 15min. Respiratory frequency and breath-to-breath irregularity scores ($irreg.score = ABS(\frac{N-(N-1)}{N})$) of frequency and amplitude (peak inspiratory airflow) were quantified during periods of resting breathing (pClamp10 software). During hypercapnic challenges, only the final 5min of the 15min period were analyzed.

Mixed brain cell culturing and staining

Using sterile technique, brains were removed from neonatal pups into ice-cold 1XHBSS (Gibco cat#14025076). Brains were washed 3X in cold PBS, then minced using surgical scissors into small pieces. These were pelleted at 100g for 5min, PBS was removed, and 0.05% trypsin/EDTA (Gibco) was added. This was at 37ºC with gentle shaking for 15-30min, with intermittent mixing and supplemental mincing, until the solution was homogenous, followed by centrifugation at 100g for 5min to pellet cells. Pellets were washed 3X with cold 1XPBS, then mixed into cell media by vigorous pipetting (recipe: 500mL of DMEM (Gibco cat#11995-065), 56.2mL of One-Shot FBS (Gibco cat#16000-077) for [c]=10%, 5.62 mL of penicillin/streptomycin, 10,000U/mL, for [c]= 100 U/mL (Gibco cat#15140122)). This was filtered through a 70-micron cell strainer (Corning) and plating onto T75 poly-d-lysine coated Nunclon flasks (Nunc EasyFlask132704) at a ratio of one brain to two flasks. After 24 hours, dead cell/debris were washed and media replaced. Cells were then incubated 24 hrs, then split onto multi-well plates with poly-d-lysine coated coverslips (Cellware 12mm coverslips) at ~25% confluence with respective treatments added from 1000X concentrated stocks in DMSO (pexidartinib) or 1XPBS (ABI-009). Media was replaced (including drugs) every 3 days, with cells fixed in cold 3.7% PFA on day 7.

Fixed coverslips were washed 3X with 1X PBS followed by a 5min incubation in 0.2% Triton-X100/1XPBS at room temperature. Slides were washed 3X in 1XPBS, blocked for 30min at room temperature in blocking solution (10% rabbit serum, 1XPBS), then incubated in antibody solution overnight at 4ºC protected from light. Antibody solution consisted of blocking solution with rabbit anti-Iba1-635 (Wako, cat#013-26471) at 1:400 and DAPI (Sigma, cat#D9542) at 10μg/mL. Data in **Figure 2** are derived from biological replicates from the same experiment, these experiments were repeated 3 times independently with similar results.

Brain immunological staining and microscopy

Brains were fixed for 48 hours in 10% formalin at 4ºC. Fixed brains were moved incubated in cryoprotection solution (30% sucrose, 1% DMSO, 100 μM glycine, 1X PBS, 0.45μm filtered, pH 7.5), and >48 hours then placed in OCT media (Tissue-Tek OCT compound, Sakura 0004348–01), frozen in cryoblock holders on dry ice, and stored at -80 until sectioned. Cryoblocks were cut at 50μm thickness using a Leica CM30505 cryostat set at -40ºC. Slices were stored in 1XPBS 4ºC until used for staining.

Slices were mounted on slides and briefly dried to adhere. Antibody staining was performed as follows: slides were first incubated in an antigen retrieval and permeabilization buffer (0.05% Triton X-100, 50μM digitonin, 10mM Tris-HCl, 1mM EDTA, pH 9.0) at 60ºC overnight in a white-light LED illuminated box (to photobleach autofluorescence). To reduce formaldehyde-induced fluorescence, slides were treated with 1mg/mL sodium borohydride in ice cold

1XPBS on ice for 30min, then moved to 10mM glycine 1XPBS, pH 7.4, for 5min at room temperature. Lipid fluorescence was blocked by incubating slides in 0.2µm filtered Sudan Black B (5mg/mL in 70% ethanol) overnight at room temperature. Slides were rinsed twice, 5min each, in 1XPBS. Excess fluid was wiped from the slide, and the tissue was circled using Liquid Blocker PAP pen (Fisher Scientific, cat#NC9827128). Slices were incubated 15min at room temperature in blocking solution (1XPBS, 10% rabbit serum (Gibco, cat#16120–099)), then stained 24hrs at 4ºC in antibody solution (rabbit anti-IBA1-fluorochrome 635 conjugated (WAKO, cat#013-26471) 1:300, mouse anti-GFAP Alexa555 conjugated (Cell Signaling, cat#3656S) 1:300, and DAPI (Sigma, cat#D9542) at 1 µg/mL) in blocking solution. Slides were washed 3X 5min in 1XPBS and mounted in aqueous mounting media (Fluoromount-G), coverslipped, and stored at 4ºC.

Staining in **Figure S9** was performed using the same method, but utilized an antibody against GFP (anti-GFP-Alexa555; Thermo Fisher, cat#A-31851, 1:300), rather than anti-GFAP.

Imaging was performed on a Zeiss LSM710 confocal microscope. Images were collected using a 10X dry objective at 0.6X optical zoom resulting in images of 1417x1417µm area. Channels were set to an optical thickness of 50nm. DAPI was excited at 405nm, with emission light collected using a sliding filter with the range setting at 424–503nm. GFAP-Alexa555 was excited with a 543nm laser, emissions collected at 548–587nm. Iba-1-635 was excited at 633nm and emitted light was collected at 641–661nm, with line averaging of 8–16 and a line scan speed of 6–7.

For quantification of lesion size, serial sectioning was performed through one half (from midline) of the brainstem. All slices were initially stained with just DAPI to allow for selection of lesion-containing sections (lesions are easily identified by hypercellular aggregation in brainstem by DAPI stain). The slice observed to have the largest lesion area was used for Iba1/GFAP staining and lesion size quantification.

For quantification of Iba1 and GFAP positive cells in a given region, an area ~10% of the total image area, with overall structure and cell composition representative of the given image, was selected and GFAP (+) and Iba1(+) cells were counted. While images were collected with identical settings, brightness and contrast were adjusted as needed to detect cell bodies during counting (we quantified cell numbers, not staining intensity, for these studies). Only cell bodies of GFAP(+) cells (astrocytes) and Iba1(+) cells (leukocytes, including microglia) were counted, with extensions from out-of-plane cells ignored. Images in main text figures have identical brightness/contrast settings; brightness of olfactory bulb images in supplemental data was adjusted to highlight stained features (there was high variability in intensity in the olfactory bulb, but this does not impact the analyses in this study as they are based on cell numbers rather than intensity).

Plasma ALT and AST measures

Small volumes (5-10µL) of blood were collected using tail-prick and heparinized microhematocrit tubes (Fisher Scientific, cat#22-362566), placed on ice in 15mL tubes, then moved (by pipette) into 1.5mL microcentrifuge tubes and stored at -80ºC until used. ALT and AST were quantified using colorimetric enzymatic activity assay kits (Sigma, cat#'s MAK052 and MAK055, respectively) according to manufacturer recommendations. Absorbance was measured on a NanoDrop1000 (ThermoScientific).

Chemokine analyses

Brainstem chemokines were analyzed by Eve Biotechnologies using the Millipore MCYTMAG-70K-PX32 Milliplex MAP Mouse Cytokine/Chemokine Magnetic Bead Panel Multiplex panel. Brainstem samples were collected rapidly after euthanasia, flash frozen in liquid nitrogen, and shipped to Eve Biotechnologies on dry ice. Samples were processed and analyzed according to Millipore manufacturer recommendations.

Drug tissue level quantification

All tissue drug level analyses were performed by the University of Washington Medicinal Chemistry Mass Spectrometry Center commercial service.

Briefly, control mice were fed drug compounded diets ad libitum until P30 (10 days, including any ramp up period, see above) to achieve steady state. Animals were euthanized in the early afternoon, tissues flash frozen. Tissues were weighed, and 3mL of 1XPBS was added while on dry ice. Samples were homogenized using an OMNI bead disruptor tissue homogenizer, chilled to -15ºC, shaken at 5.8m/s for 45s, allowed to settle for 30s, then repeated. Samples were removed and stored at -80C until extraction.

*Cal101 & IPI549

Cal101, IPI549 and glyburide (internal standard, IS) were measured using high pressure liquid chromatography-mass spectrometry (HPLC-MS). Briefly, the assays were performed on a Water's XevoTQ-XS coupled to a Water's I-Class Ultra HPLC system (Waters Corporation, Milford, MA, USA). Analytes were monitored in multiple reaction monitoring (MRM) mode selectively isolating parent m/z 416.1->176.1, 529.2->161.1 and 494.1->369.1, respectively. Chromatographic separation was achieved using a Water's Acquity T3 C18, 2.1x100mm, 1.8µ column (Waters Corporation, Milford, MA, USA). Using a gradient consisting of 0.1% formic acid (FA) in H_2O (A) and 0.1% FA in MeOH (B) at a flow rate 0.3mL/min.

10µl of IS was added to 20µl of unknown analytical sample or calibration/qc sample (10µL of stock at various concentrations + 10µL blank matrix). Samples were vortexed and 100µL of MeOH was added. Sample was vortexed again and centrifuged at 16.1rfc for 5min. 50µL of supernatant was removed and diluted with 50µL H_20 in autosampler vials, with 5µL injections.

*Pexidartinib

Pexidartinib and IS were measured using HPLC-MS. Briefly, assays were performed on a Water's Xevo TQ-XS coupled to a Water's I-Class Ultra HPLC, as above. Analytes were monitored in MRM selectively isolating parent m/z 418.2->258.1, and 494.1->369.1, respectively. Chromatographic separation was achieved using a Water's Acquity BEH C18, 2.1x50mm, 1.7µ column (Waters Corporation, Milford, MA, USA). Using a gradient consisting of 0.1% FA in H_2O (A) and 0.1% FA in acetonitrile (B) at a flow rate 0.3mL/min.

10uL of IS was added to 2µl of unknown analytical sample or calibration/qc sample (spiked into blank matrix). Samples were vortexed and 60µL of ACN was added. Sample was vortexed again and centrifuged at 16.1rfc for 5min. 40µL of supernatant was removed and diluted with 40µL of H_2O into a limited volume autosampler vial. 2µL injections were made on the platform.

Analysis of GSK2636771 and BYL719

GSK2636771, BYL719 and IS were measured using HPLC-MS. Assays were performed on a Water's Xevo TQ-XS coupled to a Water's I-Class Ultra HPLC, as above. Analytes were monitored in MRM mode selectively isolating parent m/z 434.4->215.2, 442.3->328.2 and 494.1->369.1, respectively. Chromatographic separation was achieved using a Water's Acquity BEH C18, 2.1x50mm, 1.7µ column (Waters Corporation, Milford, MA, USA). Using a gradient consisting of 0.1% formic acid in H_2O (A) and 0.1% FA in MeOH (B) at a flow rate 0.3ml/min. (can provide gradient if needed).

Blood samples: 20µL of IS (glyburide-100ng/mL) was added to 20µL of unknown analytical sample or calibration/qc samples (10uL of stock at various concentrations + 10µL of matrix). Samples were vortexed (15s) and 100µL acetonitrile added. Samples were vortexed again (15s) and centrifuged at 16.1rfc for 5min. 50µL of supernatant was removed and diluted with 50µL of H_2O in limited volume autosampler vials, 5µL injections were made.

Tissue samples: 20µL of IS (glyburide at 100ng/mL) was added to 100µL of unknown analytical sample or calibration/qc samples (50µl of stock at various concentrations + 50µL of matrix). Samples were vortexed (15s), then 1ml of 1:1 hexane: ethyl acetate was added. Samples were vortexed for 5min then centrifuged at 16.1rfc for 5min. 1mL supernatant was removed and evaporated to dryness under a stream of nitrogen. Samples were reconstituted with 50µL mobile phase A. 10µL injections were made on the platform.

Analysis was done using QuanLynxs (Waters Corporation, Milford, MA, USA) by generating a linear equation based on peak area ratios (PAR) of the analyte over the internal standard peak area. This was compiled against the expected concentration of the calibrators to generate a slope and intercept to be applied back to the PARs to obtain a return value. Acceptability of levels of quantitation was determined by a variance less than 15% from the expected return value.

For all studies a Water's Xevo TQ-XS coupled to a Water's I-Class Ultra was used in electrospray positive ionization mode (ESI-POS) with the following settings: capillary voltage (kV) – 3.1, desolvation temperature (C) – 350, desolvation gas flow – 800L/Hr N_2.

Statistical analyses

All statistical analyses were performed using GraphPad Prism as detailed in figure legends. Unless otherwise stated, error bars represent standard error of the mean (SEM) and $p<0.05$ is considered statistically significant. Unless otherwise stated, pairwise comparisons utilized unpaired, unequal variances, t-test (Welch's test). For multiple testing correction, Bonferroni corrected significance values are reported (adjusting the p-value cutoff from a starting value of 0.05), with significance assigned to individual comparisons accordingly.

Scientific rigor

Sex – approximately equal numbers of male and female animals were used in each experiment. No significant sex differences have yet been reported in the *Ndufs4*(KO) mice and none were observed here.

Sample size determination, control distribution – experiments were initially run with an n=4 animals per treatment group, providing 80% power to detect changes of 30% at a p-value of 0.05 assuming a standard deviation of 15% from the mean. Additional animals were added if variance was high, or trends toward significance were observed (to bolster or invalidate trending values). Untreated *Ndufs4*(KO)'s with advanced disease show high variance in many endpoints, necessitating increased replicate numbers. The decision to increase replicates was made when variance exceeded the aforementioned assumed SD. Datasets included no more than 2 *Ndufs4*(KO) animals from any given litter. Control treated *Ndufs4*(KO) groups are often larger for reason previously mentioned: first, variance is highest in this group; second, increasing n in this group adds to statistical power for all treatments; third, control treated animals were included throughout the course of these studies to ensure no drift in the behavior of our colony as treatment groups were studied.

Randomization and blinding – all treatment groups were randomly assigned. Blinding was performed during image analysis. Blinding of animals was often impossible (BYL719 leads to extremely small body size, pexidartinib turns fur white, etc), but experiments were scored by technicians with no expectations regarding the outcomes, and technicians trained together and cross-compared observed symptoms to ensure consistent scoring. Analysis of each group was split among the multiple technicians involved in these studies to prevent any undetected difference in scoring from differentially impacting one or more groups.

Inclusion/exclusion criteria – animals showing severe weaning stress, born as runts, or born with unrelated health issues (for example hydrocephaly) were excluded. Otherwise, no animals were excluded from randomization to treatment groups.

Supplementary Figures

A

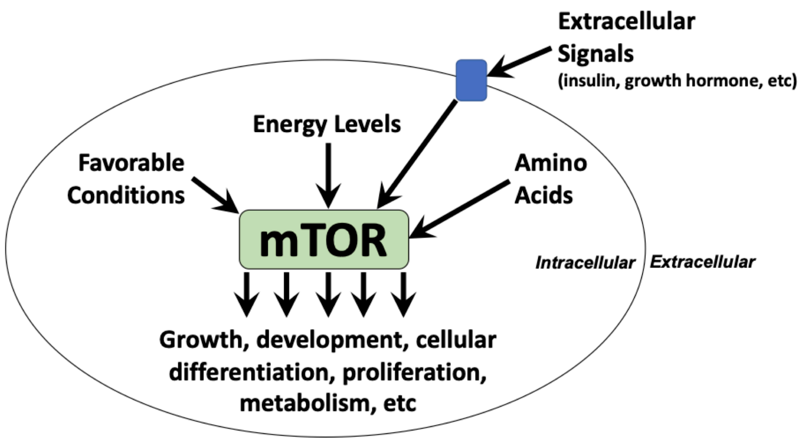

B

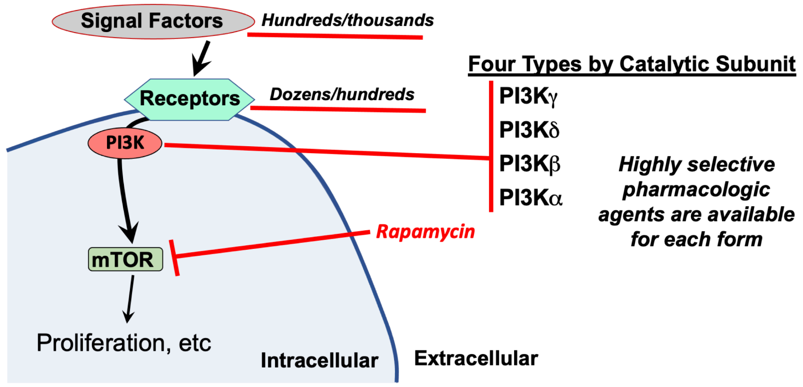

Figure S1. Probing the role of mTOR in LS. (A) mTOR is a key intracellular signaling regulator that synthesizes input from multiple upstream signaling pathways and regulates a diversity of intracellular processes involved in proliferation and growth. Upstream pathways include energy level sensing through AMPK, amino acid sensing at the lysosome, translational machinery status sensing through the ribosome, and extracellular signaling mediated by membrane bound receptors, including insulin/IGF-1 signaling, growth hormone signaling, etc. (see (8, 9) for a more detailed discussion of mTOR signaling). (2) Numerous extracellular signaling factors activate intracellular signaling through receptor/PI3K/AKT/mTOR pathways (see (8, 9)). Here, to begin probing whether the role of mTOR in LS is defined by some upstream signaling context, we chose to examine receptor/PI3K/AKT pathway upstream of mTOR. Given that there are only four catalytic subunits (p110α, p110β, p110δ, and p110γ) of PI3k, versus the dozens to hundreds of membrane bound receptors and extracellular signaling factors, we chose PI3K as a starting point for these studies. The availability of potent and highly specific orally available small molecule inhibitors of each of these catalytic subunits allowed us to utilize targeted pharmacologic screening, whereas identifying receptors or signaling factors (i.e. cytokines) will require alternate approaches.

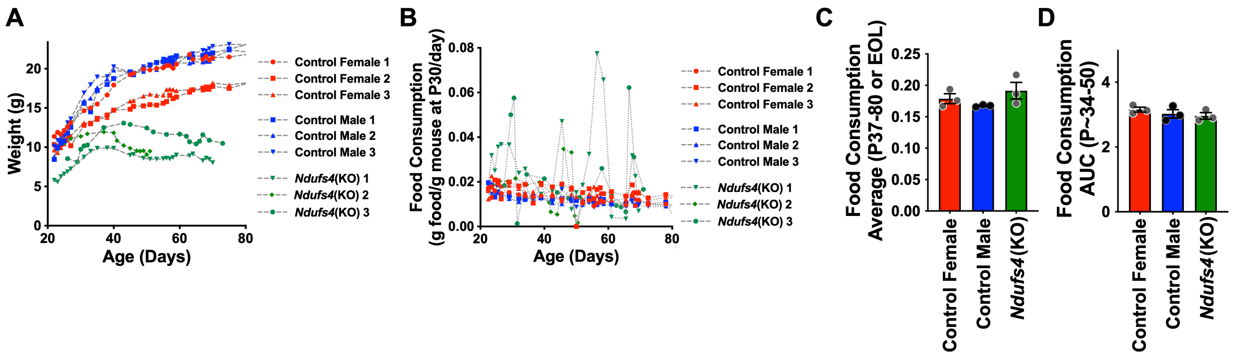

Figure S2. Cachexia occurs without anorexia in *Ndufs4*(KO). (A) Daily weight and (B) food consumption measurements for control and *Ndufs4*(KO) animals. *Ndufs4*(KO) mice are small compared to control animals, so consumption is normalized to weight at P30, a post-weaning, but pre-disease onset, weight. While there was a noticeable presence of day-to-day food consumption outlier days in the *Ndufs4*(KO) mice (B), neither the average food consumption per day (A) or total food consumption (B) throughout the period from onset of cachexia to death were different in the *Ndufs4*(KO) animals when normalizing to animal weight at P30. The age-range in (D) was set to span the period shortly prior to the onset of cachexia and continue until the first mouse was lost to euthanasia. For all panels, data represent mean, error bars ± SEM. (A-D) n's as shown.

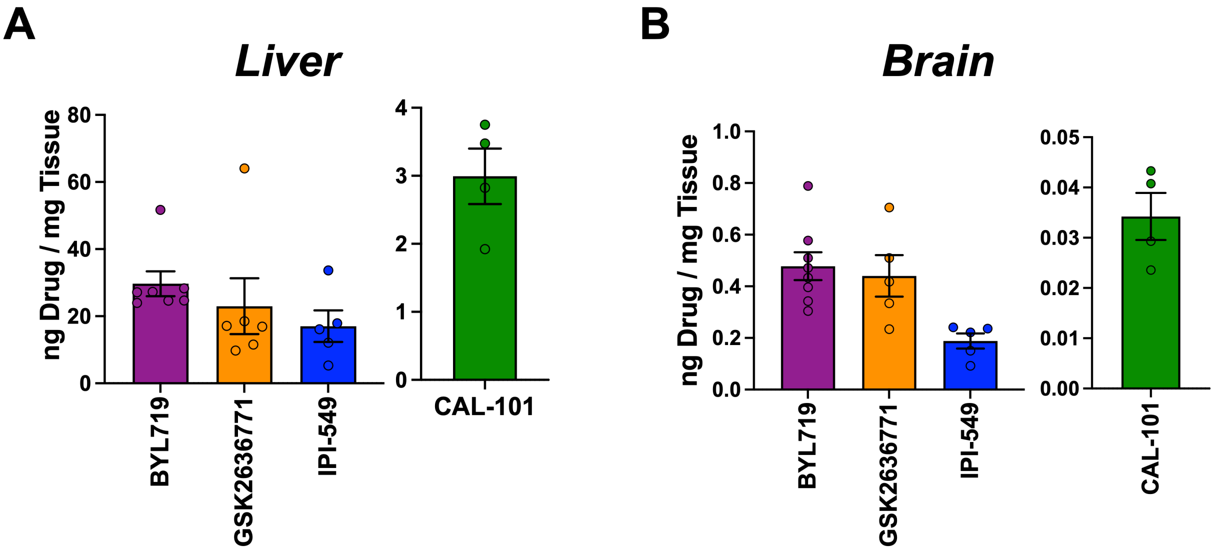

Figure S3. Tissue levels of PI3K p110 catalytic subunit isoform specific inhibitors. (A) Liver and (B) brain tissue levels of BYL719, GSK2636771, IPI-549, and CAL-101 (see **Methods**). For all panels, data represent mean, error bars ± SEM. n's as shown.

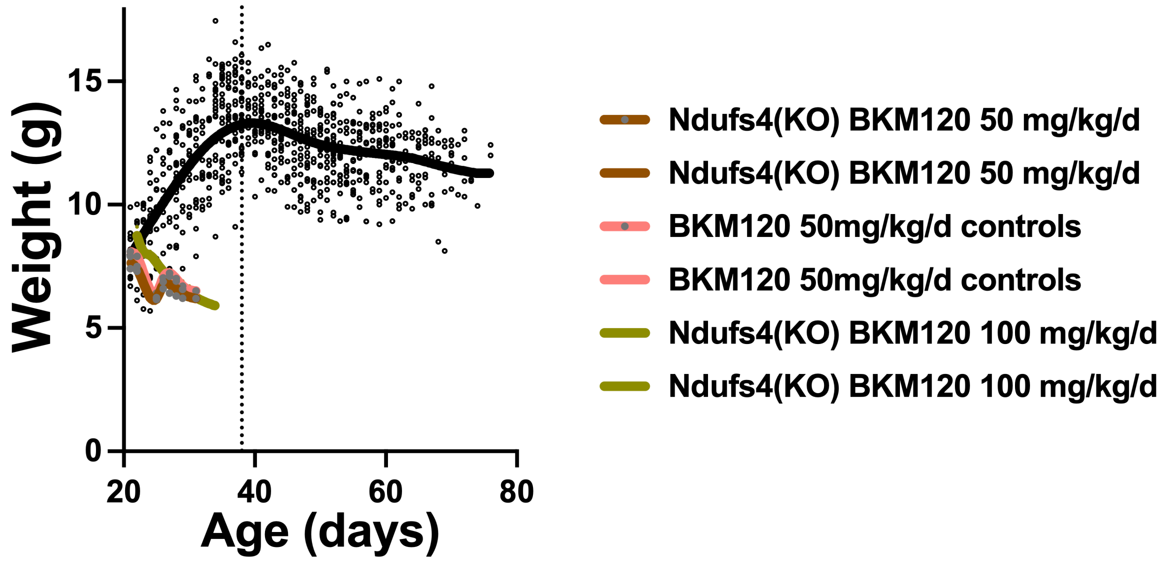

Figure S4. Impact of BKM120 during post-weaning development. BKM120 treatment at 50 or 100 mg/kg/day led to weight loss and euthanasia in both control and *Ndufs4*(KO) mice when treated during post-weaning development. n=2 animals per dose and genotype for BKM120, n for controls as in **Figure 1**. Local regression (Lowess) curve overlayed to display group trends.

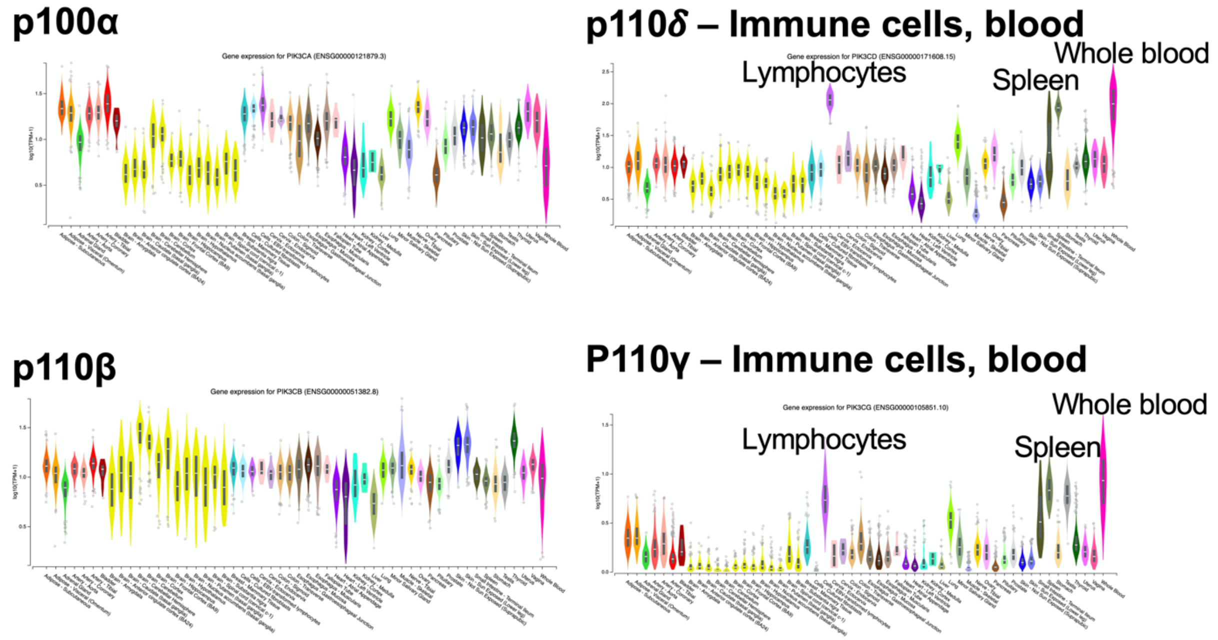

Figure S5. Expression pattern of PI3K p110 catalytic subunit isoforms. Expression of p110α, p110β, p110δ, and p110γ isoforms in different human tissues. Data and figures collected from the GTEx database by searching for PIK3CA, PIK3CB, PIK3CG, and PIK3CD, downloaded 2021. https://gtexportal.org/home/gene/ (see website for details on datasets).

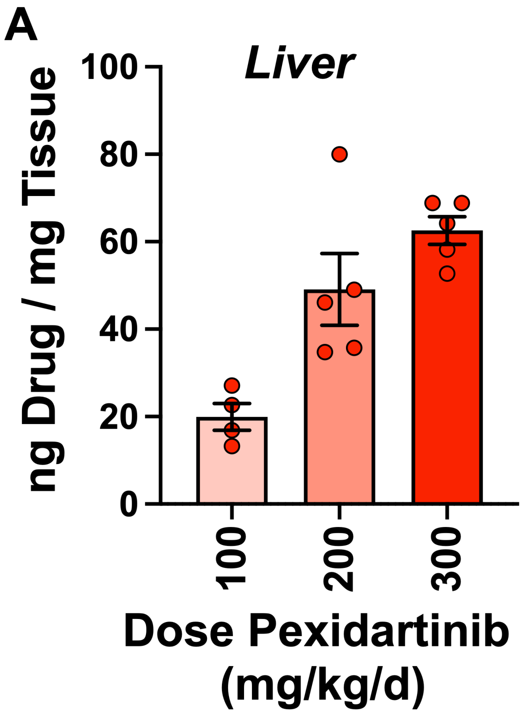

Figure S6. Tissue levels of drug in mice treated with 100, 200, or 300 mg/kg/day pexidartinib in chow. (A) Liver and (B) brain drug levels. See ***Methods***. Data represent mean, error bars ± SEM. n's as shown.

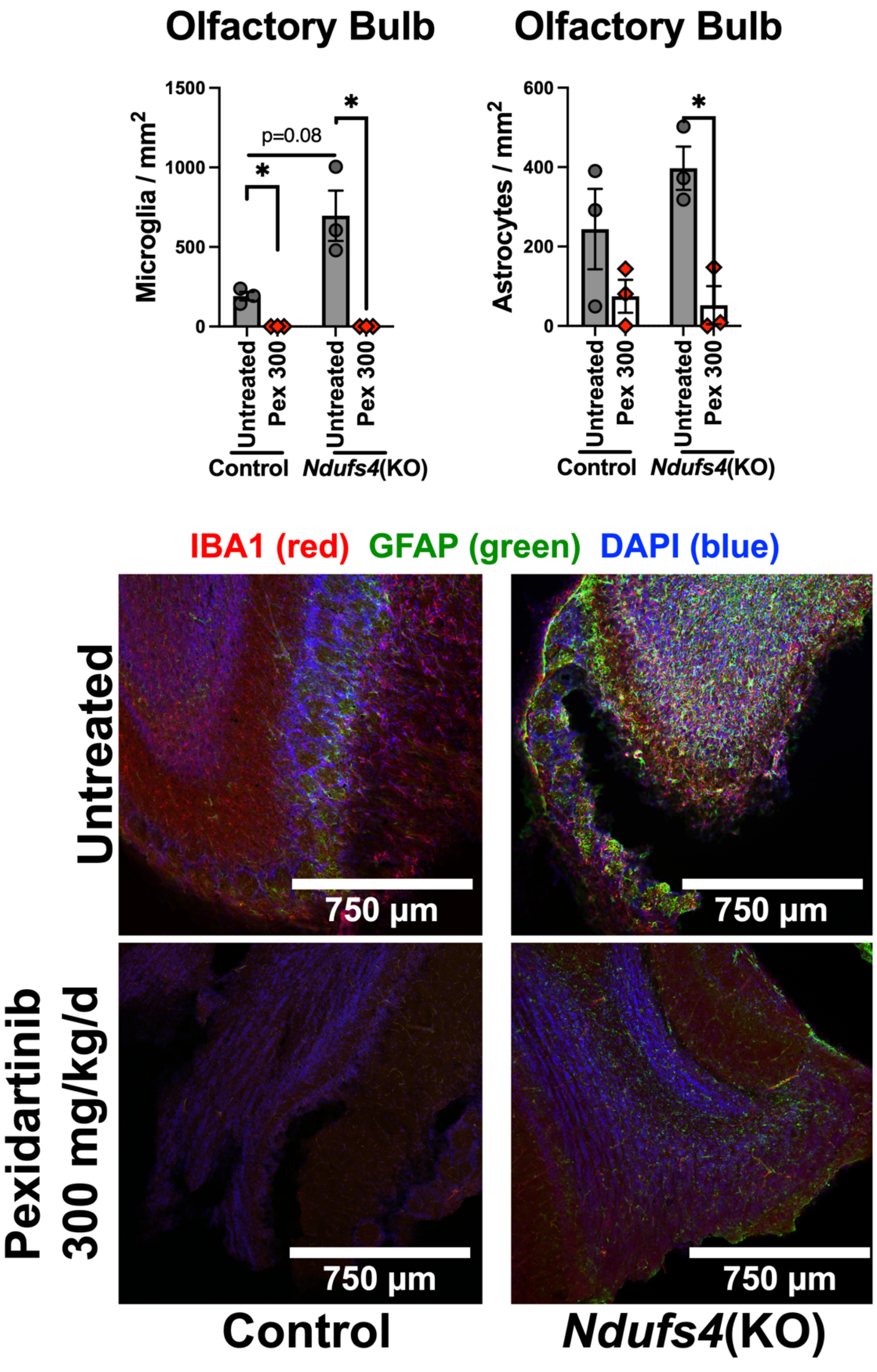

Figure S7. Impact of pexidartinib on olfactory bulb microgliosis and astrocytosis. Pexidartinib treatment appears to rescue neuroinflammation in the olfactory bulb, though high variance and limited sample numbers preclude statistical significance in some comparisons. Representative images shown, data represent mean, error bars ± SEM. n's as shown.

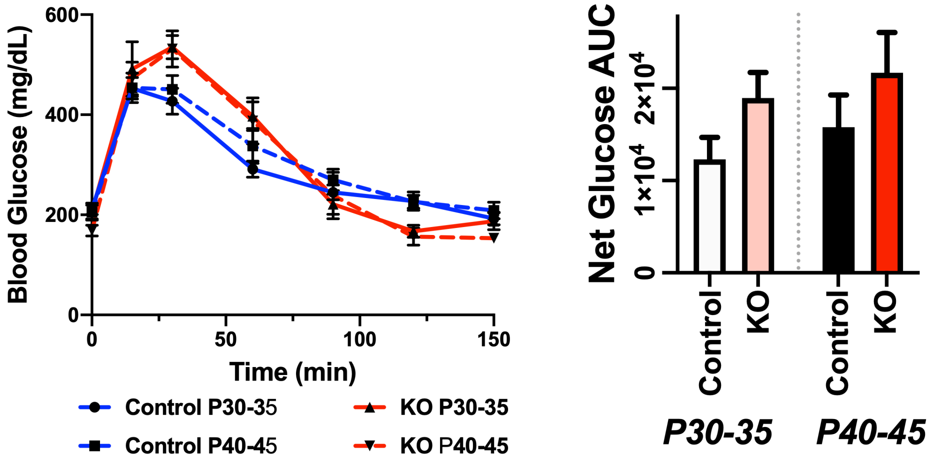

Figure S8. Glucose clearance in glucose tolerance test. Glucose clearance in the GTT assay does not change as a function of age from pre- to post- P37. Area under the curve (AUC) for glucose trends up in *Ndufs4*(KO) animals at both ages but is non-significant and the difference does not change with age. n=10 control animals per control group, 8 *Ndufs4*(KO) for each *Ndufs4*(KO) group. Data represent mean, error bars ± SEM.

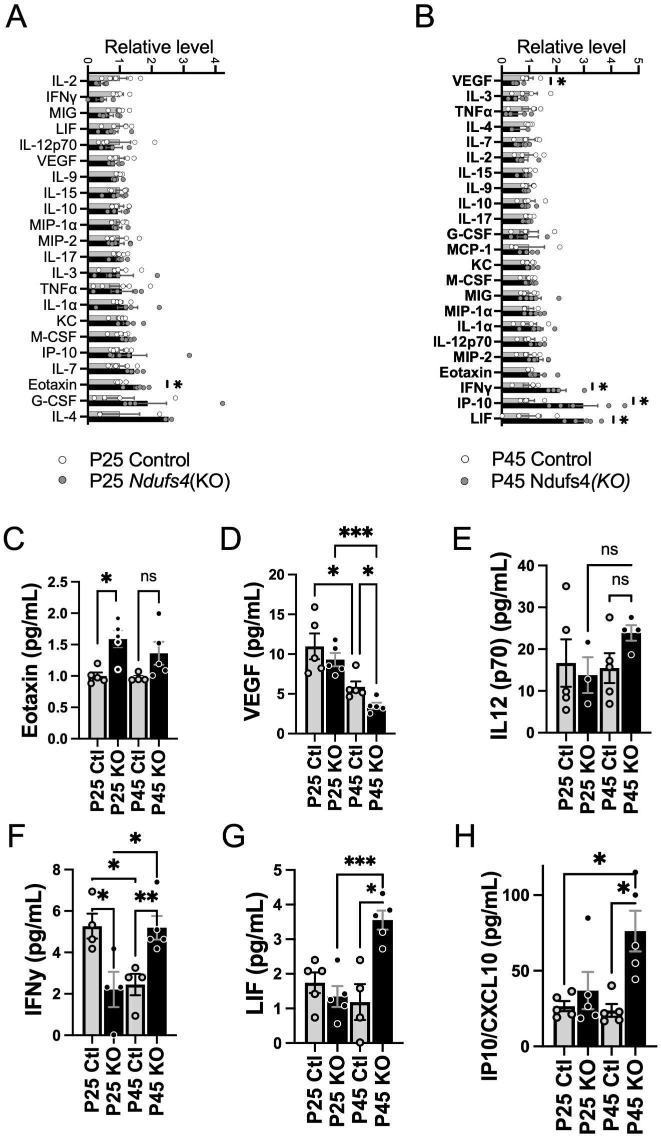

Figure S9. Targeted chemokine profiling of brainstem from pre- and post-disease onset *Ndufs4*(KO) mice. (A) Levels of 23 chemokines (see **Methods**) in brainstem of P25 *Ndufs4*(KO) and control animals. *p<0.05 by unpaired, unequal variances (Welch's) t-test. (B) Levels of 23 chemokines (see **Methods**) in brainstem of P45 *Ndufs4*(KO) and control animals. *p<0.05 by unpaired, unequal variances (Welch's) t-test. (C-H) Concentrations of select chemokines in brainstem of control and *Ndufs4*(KO) animals at P25 and P45. (C) Eotaxin (CCL11), (D) Vascular endothelial growth factor (VEGF), (E) IL12p70, (F) interferon γ (IFNγ), (G) leukemia inhibitory factor (LIF), and (H) IFNγ-induced protein 10 (IP-10). (A-H) *p<0.05, **p<0.005, and ***p<0.0005 by unpaired, unequal variances (Welch's) t-test. n=5 animals per group; in some cases individual analytes were below detection limit or failed QC, in these cases n's are lower (failed well/sample omitted).

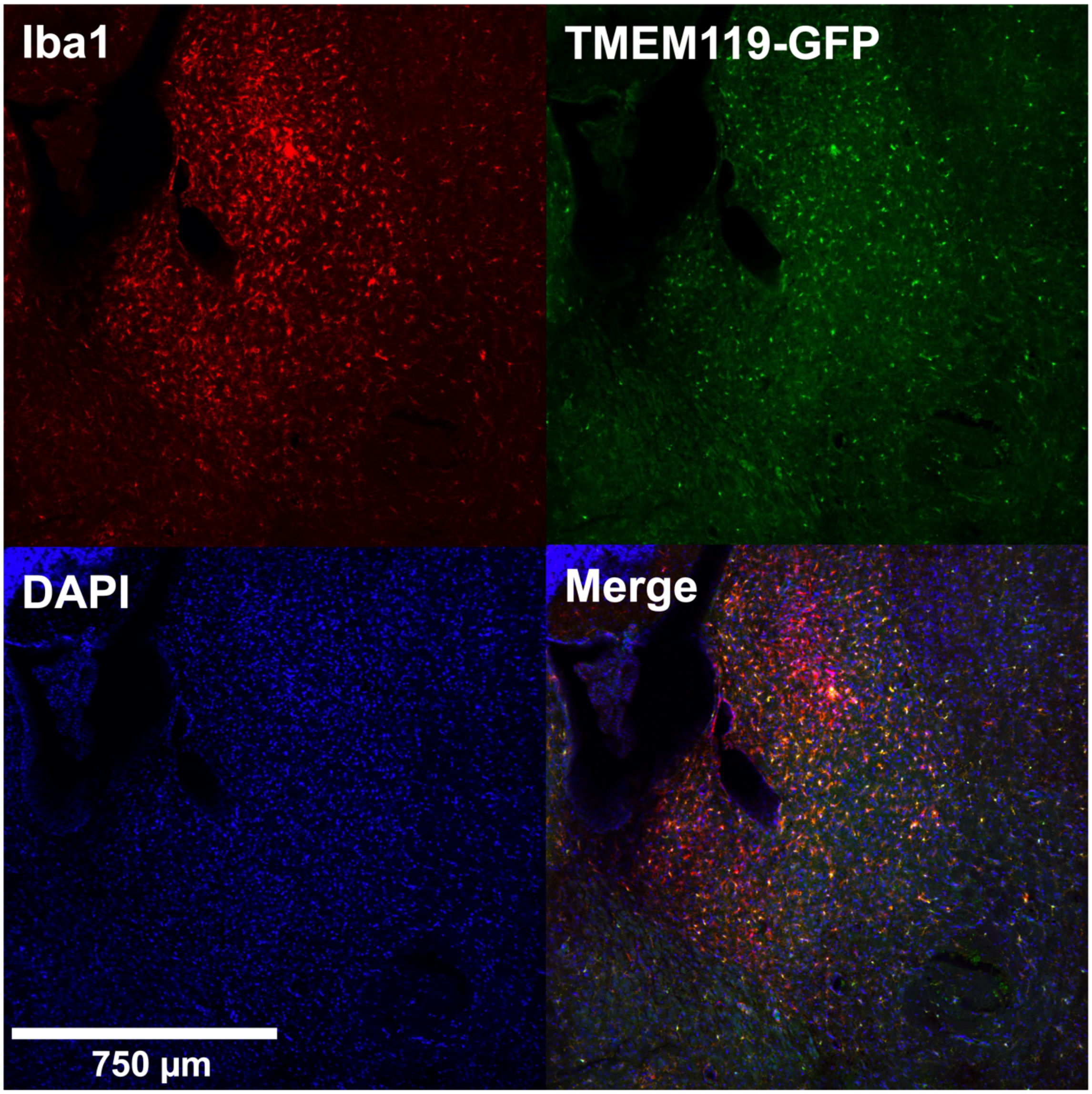

Figure S10. Peripheral leukocyte involvement in LS CNS lesions. Co-staining for GFP and Iba1 in a brainstem lesion from a TMEM-119-GFP expressing *Ndufs4*(KO) animal. The presence of Iba1(+)/TMEM-119(-) cells demonstrates, for the first time, that peripheral leukocytes participate in LS CNS lesions. Representative image of three experiments. See **Methods** for staining details.

Supplementary References

1. Johnson SC, Yanos ME, Bitto A, Castanza A, Gagnidze A, Gonzalez B, et al. Dose-dependent effects of mTOR inhibition on weight and mitochondrial disease in mice. *Front Genet.* 2015;6:247.
2. Johnson SC, Yanos ME, Kayser EB, Quintana A, Sangesland M, Castanza A, et al. mTOR inhibition alleviates mitochondrial disease in a mouse model of Leigh syndrome. *Science.* 2013;342(6165):1524-8.
3. Bornstein R, James K, Stokes J, Park KY, Kayser EB, Snell J, et al. Differential effects of mTOR inhibition and dietary ketosis in a mouse model of subacute necrotizing encephalomyelopathy. *Neurobiol Dis.* 2021;163:105594.
4. Johnson SC, Kayser EB, Bornstein R, Stokes J, Bitto A, Park KY, et al. Regional metabolic signatures in the Ndufs4(KO) mouse brain implicate defective glutamate/alpha-ketoglutarate metabolism in mitochondrial disease. *Mol Genet Metab.* 2020;130(2):118-32.
5. Johnson SC, Martinez F, Bitto A, Gonzalez B, Tazaerslan C, Cohen C, et al. mTOR inhibitors may benefit kidney transplant recipients with mitochondrial diseases. *Kidney Int.* 2019;95(2):455-66.
6. Stokes J, Freed A, Bornstein R, Su KN, Snell J, Pan A, et al. Mechanisms underlying neonate-specific metabolic effects of volatile anesthetics. *Elife.* 2021;10.
7. Sonner JM, Gong D, Li J, Eger EI, 2nd, and Laster MJ. Mouse strain modestly influences minimum alveolar anesthetic concentration and convulsivity of inhaled compounds. *Anesth Analg.* 1999;89(4):1030-4.
8. Johnson SC. Nutrient Sensing, Signaling and Ageing: The Role of IGF-1 and mTOR in Ageing and Age-Related Disease. *Subcell Biochem.* 2018;90:49-97.
9. Johnson SC, Rabinovitch PS, and Kaeberlein M. mTOR is a key modulator of ageing and age-related disease. *Nature.* 2013;493(7432):338-45.
